# Supplementary material for: Comparative efficacy and safety of traditional Chinese medicine injections in patients with transient ischemic attack: A systematic review and network meta-analysis
Source: PLoS One. 2024 Jul 24;19(7):e0307663. doi: 10.1371/journal.pone.0307663 (PMC11268667; doi:10.1371/journal.pone.0307663)
Supplement: S2 File — (DOCX) [file pone.0307663.s002.docx]

**S2 File. Search Strategy.**

**The Pubmed database Search Strategy**

#1: "Ischemic Attack, Transient"[Mesh]

Results：21899

#2: TIA[Title/Abstract] OR Transient Ischemic Attack[Title/Abstract] OR TIAs[Title/Abstract] OR Attack, Transient Ischemic[Title/Abstract] OR Attacks, Transient Ischemic[Title/Abstract] OR Ischemic Attacks, Transient[Title/Abstract] OR Transient Ischemic Attacks[Title/Abstract] OR Brain TIA[Title/Abstract] OR TIA, Brain[Title/Abstract] OR Carotid Circulation Transient Ischemic Attack[Title/Abstract] OR Transient Ischemic Attack, Carotid Circulation[Title/Abstract] OR Transient Ischemic Attack, Vertebrobasilar Circulation[Title/Abstract] OR Vertebrobasilar Circulation Transient Ischemic Attack[Title/Abstract] OR Crescendo Transient Ischemic Attacks[Title/Abstract] OR Transient Ischemic Attacks, Crescendo[Title/Abstract] OR Posterior Circulation Transient Ischemic Attack[Title/Abstract] OR Transient Ischemic Attack, Posterior Circulation[Title/Abstract] OR Transient Ischemic Attack, Anterior Circulation[Title/Abstract] OR Anterior Circulation Transient Ischemic Attack[Title/Abstract] OR Brain Stem Ischemia, Transient[Title/Abstract] OR Transient Ischemic Attack, Brain Stem[Title/Abstract] OR Brainstem Ischemia, Transient[Title/Abstract] OR Brainstem Ischemias, Transient[Title/Abstract] OR Ischemia, Transient Brainstem[Title/Abstract] OR Ischemias, Transient Brainstem[Title/Abstract] OR Transient Brainstem Ischemia[Title/Abstract] OR Brainstem Transient Ischemic Attack[Title/Abstract] OR Transient Ischemic Attack, Brainstem[Title/Abstract] OR Brain Stem Transient Ischemic Attack[Title/Abstract] OR Cerebral Ischemia, Transient[Title/Abstract] OR Cerebral Ischemias, Transient[Title/Abstract] OR Ischemia, Transient Cerebral[Title/Abstract] OR Ischemias, Transient Cerebral[Title/Abstract] OR Transient Cerebral Ischemia[Title/Abstract] OR Transient Cerebral Ischemias[Title/Abstract]

Results：33763

#3: #1 or #2

Results:42941

#4: "Injections"[Mesh]

Results：297263

#5: injection[Title/Abstract] OR injectables[Title/Abstract] OR injectable[Title/Abstract]

Results：565406

#6: #4 or #5

Results:758010

#7: randomized controlled trial[Publication Type] OR randomized[Title/Abstract] OR placebo[Title/Abstract]

Results: 1020808

#8: (#1 or #2) and (#4 or #5) and #7

Results: 83

**The Cochrane library database Search Strategy**

#1: (Ischemic Attack, Transient or TIA or Transient Ischemic Attack or TIAs or Attack, Transient Ischemic or Attacks, Transient Ischemic or Ischemic Attacks, Transient or Transient Ischemic Attacks or Brain TIA or TIA, Brain or Carotid Circulation Transient Ischemic Attack or Transient Ischemic Attack, Carotid Circulation or Transient Ischemic Attack, Vertebrobasilar Circulation or Vertebrobasilar Circulation Transient Ischemic Attack or Crescendo Transient Ischemic Attacks or Transient Ischemic Attacks, Crescendo or Posterior Circulation Transient Ischemic Attack or Transient Ischemic Attack, Posterior Circulation or Transient Ischemic Attack, Anterior Circulation or Anterior Circulation Transient Ischemic Attack or Brain Stem Ischemia, Transient or Transient Ischemic Attack, Brain Stem or Brainstem Ischemia, Transient or Brainstem Ischemias, Transient or Ischemia, Transient Brainstem or Ischemias, Transient Brainstem or Transient Brainstem Ischemia or Brainstem Transient Ischemic Attack or Transient Ischemic Attack, Brainstem or Brain Stem Transient Ischemic Attack or Cerebral Ischemia, Transient or Cerebral Ischemias, Transient or Ischemia, Transient Cerebral or Ischemias, Transient Cerebral or Transient Cerebral Ischemia or Transient Cerebral Ischemias):ti,ab,kw (Word variations have been searched) :ti,ab,kw

Results: 4943

#2: (Injections or injection or injectables or injectable):ti,ab,kw

Results: 122158

#3: #1 and #2

Results: 152

**The Embase database Search Strategy**

#1: 'transient ischemic attack':ab,ti OR 'TIA':ab,ti OR 'TIAs':ab,ti OR 'attack, transient ischemic':ab,ti OR 'attacks, transient ischemic':ab,ti OR 'ischemic attacks, transient':ab,ti OR 'transient ischemic attacks':ab,ti OR 'brain tia':ab,ti OR 'tia, brain':ab,ti OR 'carotid circulation transient ischemic attack':ab,ti OR 'transient ischemic attack, carotid circulation':ab,ti OR 'transient ischemic attack, vertebrobasilar circulation':ab,ti OR 'vertebrobasilar circulation transient ischemic attack':ab,ti OR 'crescendo transient ischemic attacks':ab,ti OR 'transient ischemic attacks, crescendo':ab,ti OR 'posterior circulation transient ischemic attack':ab,ti OR 'transient ischemic attack, posterior circulation':ab,ti OR 'transient ischemic attack, anterior circulation':ab,ti OR 'anterior circulation transient ischemic attack':ab,ti OR 'brain stem ischemia, transient':ab,ti OR 'transient ischemic attack, brain stem':ab,ti OR 'brainstem ischemia, transient':ab,ti OR 'brainstem ischemias, transient':ab,ti OR 'ischemia, transient brainstem':ab,ti OR 'ischemias, transient brainstem':ab,ti OR 'transient brainstem ischemia':ab,ti OR 'brainstem transient ischemic attack':ab,ti OR 'transient ischemic attack, brainstem':ab,ti OR 'brain stem transient ischemic attack':ab,ti OR 'cerebral ischemia, transient':ab,ti OR 'cerebral ischemias, transient':ab,ti OR 'ischemia, transient cerebral':ab,ti OR 'ischemias, transient cerebral':ab,ti OR 'transient cerebral ischemia':ab,ti OR 'transient cerebral ischemias':ab,ti

Results:62,182

#2: 'injection':ab,ti OR 'injection':ab,ti OR 'injectables':ab,ti OR 'injectable':ab,ti

Results:814,547

#3: 'randomized controlled trial':ab,ti OR 'randomized:ab,ti OR 'placebo':ab,ti

Results: 1,136,596

#4: #1 AND #2 AND #3

Results: 72

**Results of the references of included studies and reviews and Register the website of clinical trials**

References of included studied Results =0

References of reviews Results =0

Chinese Clinical Trial Registry (http://www.chictr.org.cn ) Results =0

Clinicaltrials.gov Results =0
