# Supplementary material for: Comparative efficacy and safety of traditional Chinese medicine injections in patients with transient ischemic attack: A systematic review and network meta-analysis
Source: PLoS One. 2024 Jul 24;19(7):e0307663. doi: 10.1371/journal.pone.0307663 (PMC11268667; doi:10.1371/journal.pone.0307663)
Supplement: S3 File — (DOCX) [file pone.0307663.s003.docx]

**S3 File. Details of the included TCM injections.**

Table S3 Information on Traditional Chinese Medicine Injection.

| TCM injections | Source | SFDA approval number | Scientific name of Plant or Animal | Quality control reported? (Y/N) |
| --- | --- | --- | --- | --- |
| Danhong injection | Shandong Danhong Pharmaceutical Co. | Z20026866 | Dan-Shen Root. [Lamiaceae, Salvia miltiorrhiza] (Danshen); Safflower. [Asteraceae, Carthami Flos] (Honghua). | Y^*^ |
| Xuesaitong injection | Yunnan Baiyao Group Co. | Z53021517 | pseudo-ginseng. [Araliaceae, Panax notoginseng] (Sanqi). | Y^*^ |
| Xueshuantong injection | Harbin Shengtai Biopharmaceutical Co. | Z23020823 | pseudo-ginseng. [Araliaceae, Panax notoginseng] (Sanqin). | Y^*^ |
| Dengzhanhuasu injection | Yunnan Botanical Pharmaceutical Co. | Z53020222 | Erigeron breviscapus.[Asteraceae,Erigeron breviscapus] (Dengzhanhua) | Y^*^ |
| Shuxuening injection | Shanxi Zhendong Taisheng Pharmaceutical Co. | Z14020748 | ginkgo leaf. [Ginkgoaceae,Ginkgo biloba] (Yinxing). | Y^*^ |
| Guhong injection | Tonghua Guhong Pharmaceutical Co. | H22026637 | Safflower.[Asteraceae, Carthami Flos] (Honghua). | Y^*^ |
| Shuxuetong injection | Mudanjiang Youbo Pharmaceutical Co. | Z20010100 | Leech. [.Whitmania pigra Whitman, Hirudo] (Shuizhi); Geosaurus. [Earthworm family. Pheretima] (Dilong) | Y^*^ |
| Shenxiongputao injection | Guizhou Jingfeng Injection Co. | H52020703 | Dan-Shen Root. [Lamiaceae, Salvia miltiorrhiza] (Danshen); Ligusticum wallichii. [Umbelliferae, Ligusticum chuanxiong hort] (Chuanxiong). | Y^*^ |
| Yinxingyetiquwu injection | Yuekang Pharmaceutical Group Co. | H20070226 | ginkgo leaf. [Ginkgoaceae, Ginkgo biloba] (Yinxing). | Y^*^ |

Notes: CPMs, Chinese patent medicines; SFDA, State Food and Drug Administration; *, Prepared according to People's Republic of China Pharmacopoeia.
