# Supplementary material for: Comparative efficacy and safety of traditional Chinese medicine injections in patients with transient ischemic attack: A systematic review and network meta-analysis
Source: PLoS One. 2024 Jul 24;19(7):e0307663. doi: 10.1371/journal.pone.0307663 (PMC11268667; doi:10.1371/journal.pone.0307663)
Supplement: S4 File — (DOCX) [file pone.0307663.s004.docx]

**S4 File. Risk of bias assessment.**

Figure S4 Risk of bias assessment.
