# Supplementary material for: Comparative efficacy and safety of traditional Chinese medicine injections in patients with transient ischemic attack: A systematic review and network meta-analysis
Source: PLoS One. 2024 Jul 24;19(7):e0307663. doi: 10.1371/journal.pone.0307663 (PMC11268667; doi:10.1371/journal.pone.0307663)
Supplement: S5 File — (DOCX) [file pone.0307663.s005.docx]

**S5 File. Network meta-analysis results.**

Table S5.1 League Table of total effectiveness rate.

| **Shuxuetong injection+CT** | . | . | . | . | . | . | . | . | **4.59 (3.10; 6.80)** |
| --- | --- | --- | --- | --- | --- | --- | --- | --- | --- |
| 1.02 (0.52; 2.00) | **Yinxingyetiquwu injection+CT** | . | . | . | . | . | . | . | **4.49 (2.61; 7.74)** |
| 1.02 (0.44; 2.37) | 1.00 (0.40; 2.52) | **Xuesaitong injection+CT** | . | . | . | . | . | . | **4.49 (2.13; 9.47)** |
| 1.05 (0.55; 2.02) | 1.03 (0.48; 2.18) | 1.03 (0.41; 2.55) | **Dengzhanhuasu injection+CT** | . | . | . | . | . | **4.38 (2.59; 7.38)** |
| 1.09 (0.55; 2.12) | 1.06 (0.49; 2.29) | 1.06 (0.42; 2.68) | 1.04 (0.49; 2.20) | **Danhong injection+CT** | . | . | . | . | **4.23 (2.45; 7.29)** |
| 1.12 (0.50; 2.55) | 1.10 (0.45; 2.71) | 1.10 (0.39; 3.10) | 1.07 (0.44; 2.60) | 1.03 (0.42; 2.55) | **Guhong injection+CT** | **.** | . | . | **4.09 (1.99; 8.39)** |
| 1.33 (0.65; 2.73) | 1.30 (0.58; 2.93) | 1.30 (0.50; 3.40) | 1.27 (0.57; 2.82) | 1.22 (0.54; 2.76) | 1.18 (0.46; 3.03) | **Shenxiongputao injection+CT** | . | . | **3.45 (1.89; 6.32)** |
| 1.44 (0.63; 3.32) | 1.41 (0.57; 3.52) | 1.41 (0.50; 4.02) | 1.38 (0.56; 3.39) | 1.33 (0.53; 3.32) | 1.28 (0.46; 3.59) | 1.08 (0.42; 2.81) | **Shuxuening injection+CT** | . | **3.18 (1.53; 6.64)** |
| 1.56 (0.61; 4.04) | 1.53 (0.55; 4.25) | 1.53 (0.49; 4.79) | 1.49 (0.54; 4.09) | 1.44 (0.52; 4.00) | 1.39 (0.45; 4.29) | 1.18 (0.41; 3.38) | 1.08 (0.35; 3.37) | **Xueshuantong injection+CT** | **2.93 (1.24; 6.96)** |
| **4.59 (3.10; 6.80)** | **4.49 (2.61; 7.74)** | **4.49 (2.13; 9.47)** | **4.38 (2.59; 7.38)** | **4.23 (2.45; 7.29)** | **4.09 (1.99; 8.39)** | **3.45 (1.89; 6.32)** | **3.18 (1.53; 6.64)** | **2.93 (1.24; 6.96)** | **CT** |

All results are presented in the form of OR (95% CI). Different traditional Chinese injections are ranked according to the surface under the curve cumulative for total effectiveness rate starting with the best from left to right. The results of the network meta-analysis are showed in the lower left part, and results from pairwise comparisons in the upper right half (if available). Cells shown in bold indicate significant results. CT, conventional treatment.

Table S5.2 League Table of plasma viscosity.

| **Dengzhanhuasu injection+CT** | . | . | . | . | . | . | . | **-1.09 (-1.43; -0.76)** |
| --- | --- | --- | --- | --- | --- | --- | --- | --- |
| **-0.73 (****-1.10; -0.35)** | **Yinxingyetiquwu injection+CT** | . | . | . | . | . | . | **-0.37 (-0.53; -0.20)** |
| **-0.74 (****-1.14; -0.33)** | -0.01 (-0.29; 0.27) | **Shenxiongputao injection+CT** | . | . | . | . | . | **-0.36 (-0.58; -0.14)** |
| **-0.73 (****-1.19; -0.27)** | -0.01 (-0.37; 0.36) | 0.00 (-0.39; 0.39) | **Guhong injection+CT** | . | . | . | . | **-0.36 (-0.68; -0.04)** |
| **-0.86 (****-1.33; -0.38)** | -0.13 (-0.51; 0.25) | -0.12 (-0.52; 0.28) | -0.12 (-0.59; 0.34) | **Danhong injection+CT** | . | . | . | -0.24 (-0.57; 0.10) |
| **-0.89 (****-1.29; -0.48)** | -0.16 (-0.45; 0.12) | -0.15 (-0.47; 0.17) | -0.16 (-0.55; 0.24) | -0.03 (-0.44; 0.38) | **Shuxuetong injection+CT** | . | . | -0.20 (-0.44; 0.03) |
| **-0.90 (****-1.48; -0.33)** | -0.18 (-0.67; 0.32) | -0.17 (-0.68; 0.35) | -0.17 (-0.74; 0.39) | -0.05 (-0.62; 0.53) | -0.01 (-0.53; 0.51) | **Xuesaitong injection+CT** | . | -0.19 (-0.66; 0.28) |
| **-0.95 (****-1.42; -0.49)** | -0.23 (-0.59; 0.13) | -0.22 (-0.61; 0.17) | -0.22 (-0.68; 0.23) | -0.10 (-0.56; 0.37) | -0.07 (-0.46; 0.33) | -0.05 (-0.62; 0.51) | **Shuxuening injection+CT** | -0.14 (-0.46; 0.18) |
| **-1.09 (****-1.43; -0.76)** | **-0.37 (****-0.53; -0.20)** | **-0.36 (****-0.58; -0.14)** | **-0.36 (****-0.68; -0.04)** | -0.24 (-0.57; 0.10) | -0.20 (-0.44; 0.03) | -0.19 (-0.66; 0.28) | -0.14 (-0.46; 0.18) | **CT** |

All results are presented in the form of MD (95% CI). Different traditional Chinese injections are ranked according to the surface under the curve cumulative for plasma viscosity starting with the best from left to right. The results of the network meta-analysis are showed in the lower left part, and results from pairwise comparisons in the upper right half (if available). Cells shown in bold indicate significant results. CT, conventional treatment.

Table S5.3 League Table of fibrinogen.

| **Shuxuetong injection+CT** | . | . | . | . | . | . | . | -1.83 (-2.77; -0.88) |
| --- | --- | --- | --- | --- | --- | --- | --- | --- |
| -0.51 (-1.86; 0.83) | **Dengzhanhuasu injection+CT** | . | . | . | . | . | . | **-1.31 (-2.26; -0.36)** |
| -0.62 (-2.29; 1.05) | -0.10 (-1.77; 1.57) | **Xuesaitong injection+CT** | . | . | . | . | . | -1.21 (-2.58; 0.16) |
| -0.78 (-2.12; 0.56) | -0.26 (-1.60; 1.08) | -0.16 (-1.83; 1.51) | **Guhong injection+CT** | . | . | . | . | **-1.05 (-2.00; -0.10)** |
| -0.92 (-2.09; 0.25) | -0.41 (-1.58; 0.76) | -0.30 (-1.84; 1.23) | -0.15 (-1.32; 1.03) | **Shenxiongputao injection+CT** | . | . | . | **-0.91 (-1.59; -0.22)** |
| **-1.12 (****-2.21; -0.02)** | -0.60 (-1.70; 0.50) | -0.50 (-1.98; 0.99) | -0.34 (-1.44; 0.76) | -0.19 (-1.08; 0.69) | **Yinxingyetiquwu injection+CT** | . | . | **-0.71 (-1.27; -0.16)** |
| -1.22 (-2.86; 0.42) | -0.70 (-2.35; 0.94) | -0.60 (-2.52; 1.32) | -0.44 (-2.08; 1.20) | -0.30 (-1.80; 1.21) | -0.10 (-1.56; 1.35) | **Shuxuening injection+CT** | . | -0.61 (-1.95; 0.73) |
| **-1.34 (****-2.50; -0.18)** | -0.83 (-1.99; 0.34) | -0.72 (-2.25; 0.81) | -0.56 (-1.72; 0.60) | -0.42 (-1.38; 0.55) | -0.22 (-1.10; 0.65) | -0.12 (-1.62; 1.38) | **Xueshuantong injection+CT** | -0.49 (-1.16; 0.18) |
| **-1.83 (****-2.77; -0.88)** | **-1.31 (****-2.26; -0.36)** | -1.21 (-2.58; 0.16) | **-1.05 (****-2.00; -0.10)** | **-0.91 (****-1.59; -0.22)** | **-0.71 (****-1.27; -0.16)** | -0.61 (-1.95; 0.73) | -0.49 (-1.16; 0.18) | **CT** |

All results are presented in the form of MD (95% CI). Different traditional Chinese injections are ranked according to the surface under the curve cumulative for fibrinogen starting with the best from left to right. The results of the network meta-analysis are showed in the lower left part, and results from pairwise comparisons in the upper right half (if available). Cells shown in bold indicate significant results. CT, conventional treatment.

Table S5.4 League Table of whole blood reduced viscosity (high shear rate).

| **Dengzhanhuasu injection+CT** | . | . | . | . | . | . | . | **-1.26 (-1.67; -0.85)** |
| --- | --- | --- | --- | --- | --- | --- | --- | --- |
| -0.08 (-0.66; 0.50) | **Yinxingyetiquwu injection+CT** | . | . | . | . | . | . | **-1.18 (-1.58; -0.78)** |
| -0.23 (-1.03; 0.58) | -0.15 (-0.95; 0.65) | **Xuesaitong injection+CT** | . | . | . | . | . | **-1.03 (-1.72; -0.34)** |
| -0.28 (-0.90; 0.33) | -0.20 (-0.81; 0.40) | -0.05 (-0.88; 0.77) | **Guhong injection+CT** | . | . | . | . | **-0.98 (-1.43; -0.52)** |
| -0.36 (-0.97; 0.25) | -0.28 (-0.88; 0.32) | -0.13 (-0.95; 0.69) | -0.07 (-0.71; 0.56) | **Shuxuening injection+CT** | . | . | . | **-0.90 (-1.35; -0.46)** |
| -0.48 (-1.06; 0.11) | -0.40 (-0.98; 0.18) | -0.25 (-1.05; 0.56) | -0.19 (-0.81; 0.42) | -0.12 (-0.73; 0.49) | **Danhong injection+CT** | . | . | **-0.78 (-1.20; -0.37)** |
| **-0.62 (****-1.12; -0.12)** | **-0.54 (****-1.03; -0.05)** | -0.39 (-1.13; 0.36) | -0.33 (-0.86; 0.20) | -0.26 (-0.78; 0.27) | -0.14 (-0.64; 0.36) | **Shenxiongputao injection+CT** | . | **-0.64 (-0.92; -0.36)** |
| **-0.75 (****-1.23; -0.27)** | **-0.67 (****-1.14; -0.20)** | -0.52 (-1.25; 0.21) | -0.47 (-0.98; 0.05) | -0.39 (-0.90; 0.12) | -0.27 (-0.75; 0.21) | -0.13 (-0.50; 0.24) | **Shuxuetong injection+CT** | **-0.51 (-0.75; -0.26)** |
| **-1.26 (****-1.67; -0.85)** | **-1.18 (****-1.58; -0.78)** | **-1.03 (****-1.72; -0.34)** | **-0.98 (****-1.43; -0.52)** | **-0.90 (****-1.35; -0.46)** | **-0.78 (****-1.20; -0.37)** | **-0.64 (****-0.92; -0.36)** | **-0.51 (****-0.75; -0.26)** | **CT** |

All results are presented in the form of MD (95% CI). Different traditional Chinese injections are ranked according to the surface under the curve cumulative for whole blood reduced viscosity (high shear rate) starting with the best from left to right. The results of the network meta-analysis are showed in the lower left part, and results from pairwise comparisons in the upper right half (if available). Cells shown in bold indicate significant results. CT, conventional treatment.

Table S5.5 League Table of whole blood reduced viscosity (low shear rate).

| **Dengzhanhuasu injection+CT** | . | . | . | . | . | . | . | **-2.81 (-4.58; -1.05)** |
| --- | --- | --- | --- | --- | --- | --- | --- | --- |
| -0.48 (-2.72; 1.76) | **Shuxuening injection+CT** | . | . | . | . | . | . | **-2.34 (-3.71; -0.96)** |
| -1.25 (-3.51; 1.02) | -0.77 (-2.75; 1.22) | **Danhong injection+CT** | . | . | . | . | . | **-1.57 (-3.00; -0.14)** |
| -1.58 (-4.31; 1.14) | -1.11 (-3.60; 1.39) | -0.34 (-2.86; 2.18) | **Xuesaitong injection+CT** | . | . | . | . | -1.23 (-3.31; 0.85) |
| -1.63 (-3.83; 0.57) | -1.15 (-3.06; 0.76) | -0.38 (-2.33; 1.56) | -0.05 (-2.51; 2.42) | **Yinxingyetiquwu injection+CT** | . | . | . | -1.18 (-2.51; 0.14) |
| -1.64 (-3.62; 0.35) | -1.16 (-2.81; 0.49) | -0.39 (-2.08; 1.30) | -0.05 (-2.32; 2.22) | -0.01 (-1.61; 1.60) | **Shenxiongputao injection+CT** | . | . | **-1.18 (-2.09; -0.27)** |
| -1.70 (-3.71; 0.30) | -1.23 (-2.90; 0.45) | -0.46 (-2.18; 1.26) | -0.12 (-2.41; 2.17) | -0.07 (-1.71; 1.56) | -0.07 (-1.39; 1.25) | **Shuxuetong injection+CT** | . | **-1.11 (-2.06; -0.16)** |
| **-2.39 (****-4.63; -0.15)** | -1.91 (-3.86; 0.04) | -1.14 (-3.13; 0.84) | -0.80 (-3.30; 1.69) | -0.76 (-2.67; 1.15) | -0.75 (-2.40; 0.90) | -0.68 (-2.36; 0.99) | **Guhong injection+CT** | -0.43 (-1.80; 0.95) |
| **-2.81 (****-4.58; -1.05)** | **-2.34 (****-3.71; -0.96)** | **-1.57 (****-3.00; -0.14)** | -1.23 (-3.31; 0.85) | -1.18 (-2.51; 0.14) | **-1.18 (****-2.09; -0.27)** | **-1.11 (****-2.06; -0.16)** | -0.43 (-1.80; 0.95) | **CT** |

All results are presented in the form of MD (95% CI). Different traditional Chinese injections are ranked according to the surface under the curve cumulative for whole blood reduced viscosity (low shear rate) starting with the best from left to right. The results of the network meta-analysis are showed in the lower left part, and results from pairwise comparisons in the upper right half (if available). Cells shown in bold indicate significant results. CT, conventional treatment.

Table S5.6 League Table of total cholesterol.

| **Shuxuetong injection+CT** | . | . | . | **-5.79 (-6.22; -5.36)** |
| --- | --- | --- | --- | --- |
| **-4.25 (-4.94; -3.56)** | **Danhong injection+CT** | . | . | **-1.54 (-2.08; -1.00)** |
| **-4.29 (-5.04; -3.54)** | -0.04 (-0.86; 0.78) | **Shuxuening injection+CT** | . | **-1.50 (-2.11; -0.89)** |
| **-4.89 (****-5.35; -4.43)** | **-0.64 (-1.20; -0.08)** | -0.60 (-1.24; 0.04) | **Xueshuantong injection+CT** | **-0.90 (-1.07; -0.73)** |
| **-5.79 (-6.22; -5.36)** | **-1.54 (-2.08; -1.00)** | **-1.50 (-2.11; -0.89)** | **-0.90 (-1.07; -0.73)** | **CT** |

All results are presented in the form of MD (95% CI). Different traditional Chinese injections are ranked according to the surface under the curve cumulative for total cholesterol starting with the best from left to right. The results of the network meta-analysis are showed in the lower left part, and results from pairwise comparisons in the upper right half (if available). Cells shown in bold indicate significant results. CT, conventional treatment.

Table S5.7 Table of triglyceride.

| **Shuxuetong injection+CT** | . | **-2.55 (-2.90; -2.20)** | . |
| --- | --- | --- | --- |
| **-2.05 (****-2.41; -1.69)** | **Xueshuantong injection+CT** | **-0.50 (-0.58; -0.42)** | . |
| **-2.55 (****-2.90; -2.20)** | **-0.50 (****-0.58; -0.42)** | **CT** | -0.01 (-0.22; 0.20) |
| **-2.56 (****-2.97; -2.15)** | **-0.51 (****-0.74; -0.29)** | -0.01 (-0.22; 0.20) | **Danhong injection+CT** |

All results are presented in the form of MD (95% CI). Different traditional Chinese injections are ranked according to the surface under the curve cumulative for triglyceride starting with the best from left to right. The results of the network meta-analysis are showed in the lower left part, and results from pairwise comparisons in the upper right half (if available). Cells shown in bold indicate significant results. CT, conventional treatment.

Table S5.8 Table of incidence of Cerebral Infarction.

| **Yinxingyetiquwu injection+CT** | . | . | . | . | . | . | 0.13 (0.01; 1.06) |
| --- | --- | --- | --- | --- | --- | --- | --- |
| 0.67 (0.06; 7.11) | **Shuxuetong injection+CT** | . | . | . | . | . | **0.19 (0.07; 0.51)** |
| 0.68 (0.04; 10.97) | 1.01 (0.13; 7.84) | **Xuesaitong injection+CT** | . | . | . | . | 0.19 (0.03; 1.10) |
| 0.62 (0.03; 13.15) | 0.92 (0.08; 10.24) | 0.91 (0.05; 15.26) | **Dengzhanhuasu injection+CT** | . | . | . | 0.20 (0.02; 1.82) |
| 0.57 (0.05; 6.44) | 0.84 (0.18; 3.93) | 0.83 (0.10; 6.99) | 0.92 (0.08; 10.96) | **Danhong injection+CT** | . | . | **0.22 (0.07; 0.71)** |
| 0.40 (0.02; 6.87) | 0.59 (0.07; 5.02) | 0.59 (0.04; 7.82) | 0.64 (0.04; 11.59) | 0.70 (0.08; 6.42) | **Shuxuening injection+CT** | . | 0.32 (0.05; 2.08) |
| 0.31 (0.03; 3.74) | 0.46 (0.09; 2.37) | 0.45 (0.05; 4.10) | 0.50 (0.04; 6.35) | 0.54 (0.09; 3.10) | 0.77 (0.08; 7.62) | **Shenxiongputao injection+CT** | 0.41 (0.11; 1.52) |
| 0.13 (0.01; 1.06) | **0.19 (****0.07; 0.51)** | 0.19 (0.03; 1.10) | 0.20 (0.02; 1.82) | **0.22** **(0.07; 0.71)** | 0.32 (0.05; 2.08) | 0.41 (0.11; 1.52) | **CT** |

All results are presented in the form of MD (95% CI). Different traditional Chinese injections are ranked according to the surface under the curve cumulative for incidence of Cerebral Infarction starting with the best from left to right. The results of the network meta-analysis are showed in the lower left part, and results from pairwise comparisons in the upper right half (if available). Cells shown in bold indicate significant results. CT, conventional treatment.
