# Supplementary material for: Comparative efficacy and safety of traditional Chinese medicine injections in patients with transient ischemic attack: A systematic review and network meta-analysis
Source: PLoS One. 2024 Jul 24;19(7):e0307663. doi: 10.1371/journal.pone.0307663 (PMC11268667; doi:10.1371/journal.pone.0307663)
Supplement: S6 File — (DOCX) [file pone.0307663.s006.docx]

**S6 File. Evaluation of heterogeneity and inconsistency.**

**Table 6.1 Evaluation of heterogeneity.**

| Outcomes | Number | Heterogeneity | | | | | Heterogeneity  assessment |
| --- | --- | --- | --- | --- | --- | --- | --- |
|  |  | τ^2^ | Q | df | *P* | I^2^ (95% CI) |  |
| Total effectiveness rate | 48 | 0 | 14.87 | 39 | 0.9998 | 0% | low |
| Plasma viscosity | 24 | 0.05 | 564.3 | 16 | < 0.0001 | 97.2% | high |
| Fibrinogen | 22 | 0.4603 | 539.52 | 14 | < 0.0001 | 97.4% | high |
| Whole blood reduced viscosity (high shear rate) | 20 | 0.0698 | 128.71 | 12 | < 0.0001 | 90.7% | high |
| Whole blood reduced viscosity (low shear rate) | 19 | 0.8305 | 185.96 | 11 | < 0.0001 | 94.1% | high |
| Total cholesterol | 8 | 0 | 0.35 | 4 | 0.9863 | 0% | low |
| Triglyceride | 7 | 0.0001 | 4.02 | 4 | 0.4032 | 0.5% | low |
| Incidence of cerebral infarction | 17 | 0 | 2.41 | 10 | 0.9921 | 0% | low |

**Podal split analyses of the network meta-analysis.**

**①Total effective rate.②Plasma viscosity；③Fibrinogen； ④Whole blood reduced viscosity (high shear rate)；⑤Whole blood reduced viscosity (low shear rate); ⑥****TC; ⑦TG; ⑧Incidence of cerebral infarction.**


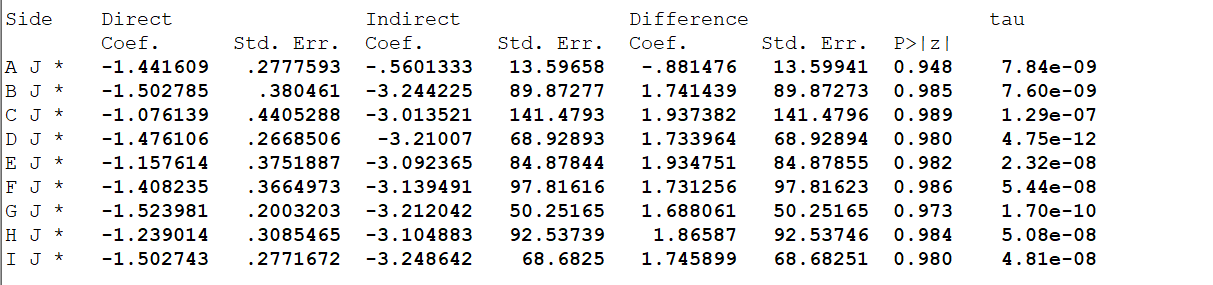


1. Nodal split analyses with total effective rate. A, Danhong injection+CT. B, Xuesaitong injection+CT. C, Xueshuantong injection+CT. D, Degzhanhuasu injection+CT. E, Shuxuening injection+CT. F, Guhong injection+CT. G, Shuxuetong injection+CT. H, Shenxiongputao injection+CT. I, Yinxingyetiquwu injection+CT. J, CT.


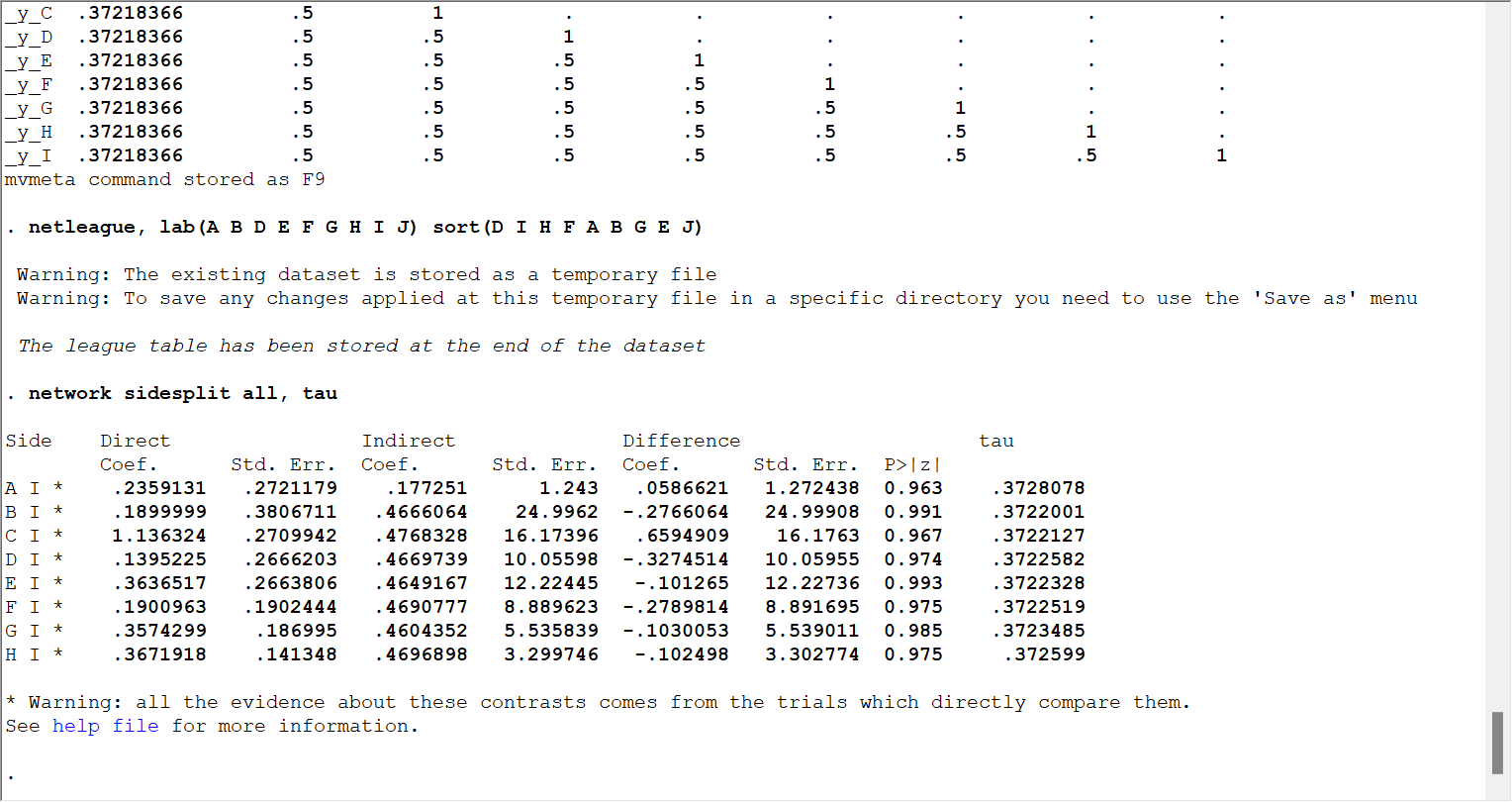


1. Nodal split analyses with plasma viscosity. A, Danhong injection+CT. B, Xuesaitong injection+CT. C, Dengzhanhuasu injection+CT. D, Shuxuening injection+CT. E, Guhong injection+CT. F, Shuxuetong injection+CT. G, Shenxiongputao injection+CT. H, Yinxingyetiquwu injection+CT. I, CT.


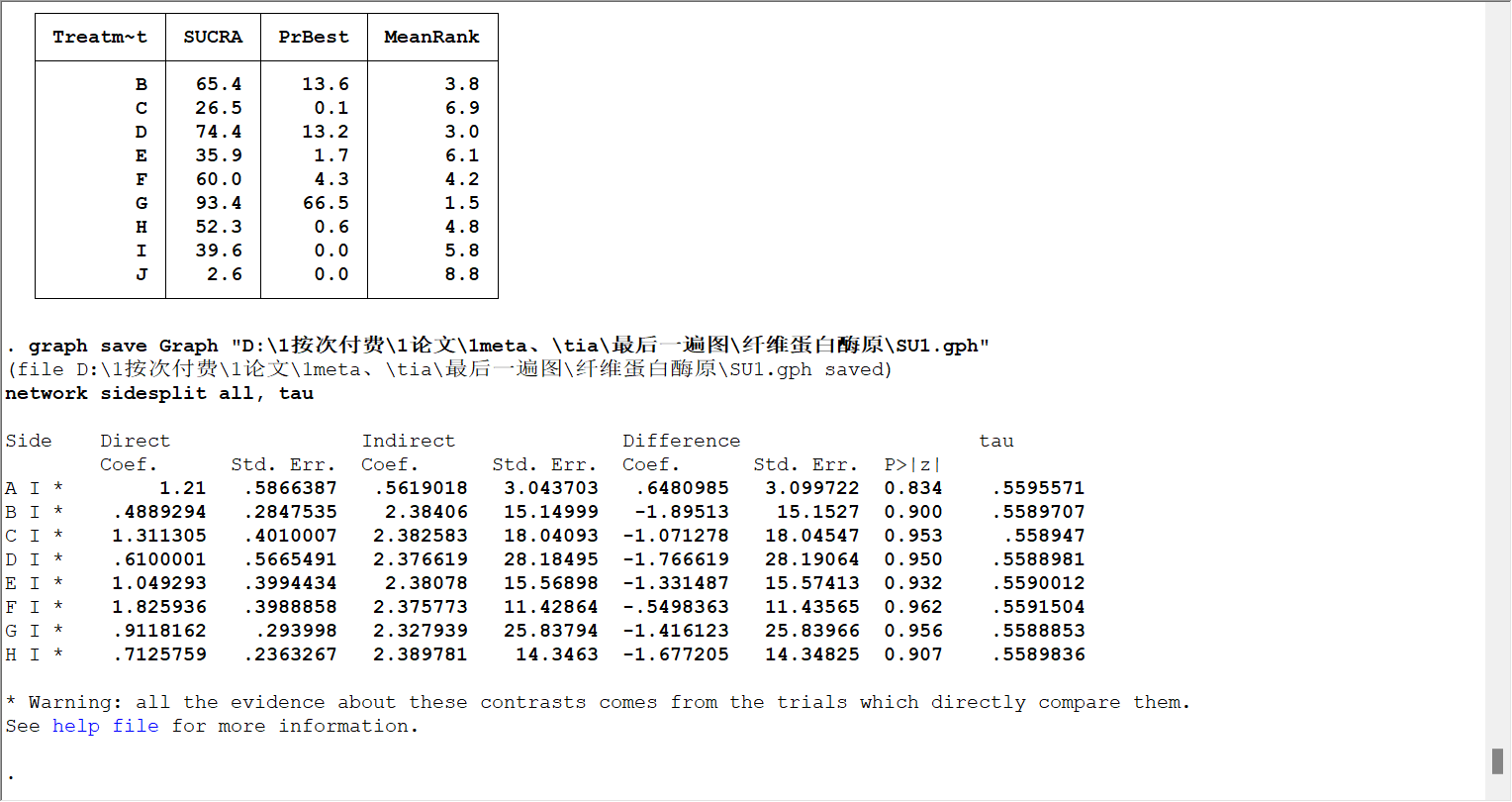


1. Nodal split analyses with fibrinogen. A, Xuesaitong injection+CT. B, Xueshuantong injection+CT. C, Dengzhanhuasu injection+CT. D, Shuxuening injection+CT. E, Guhong injection+CT. F, Shuxuetong injection+CT. G, Shenxiongputao injection+CT. H, Yinxingyetiquwu injection+CT. I, CT.


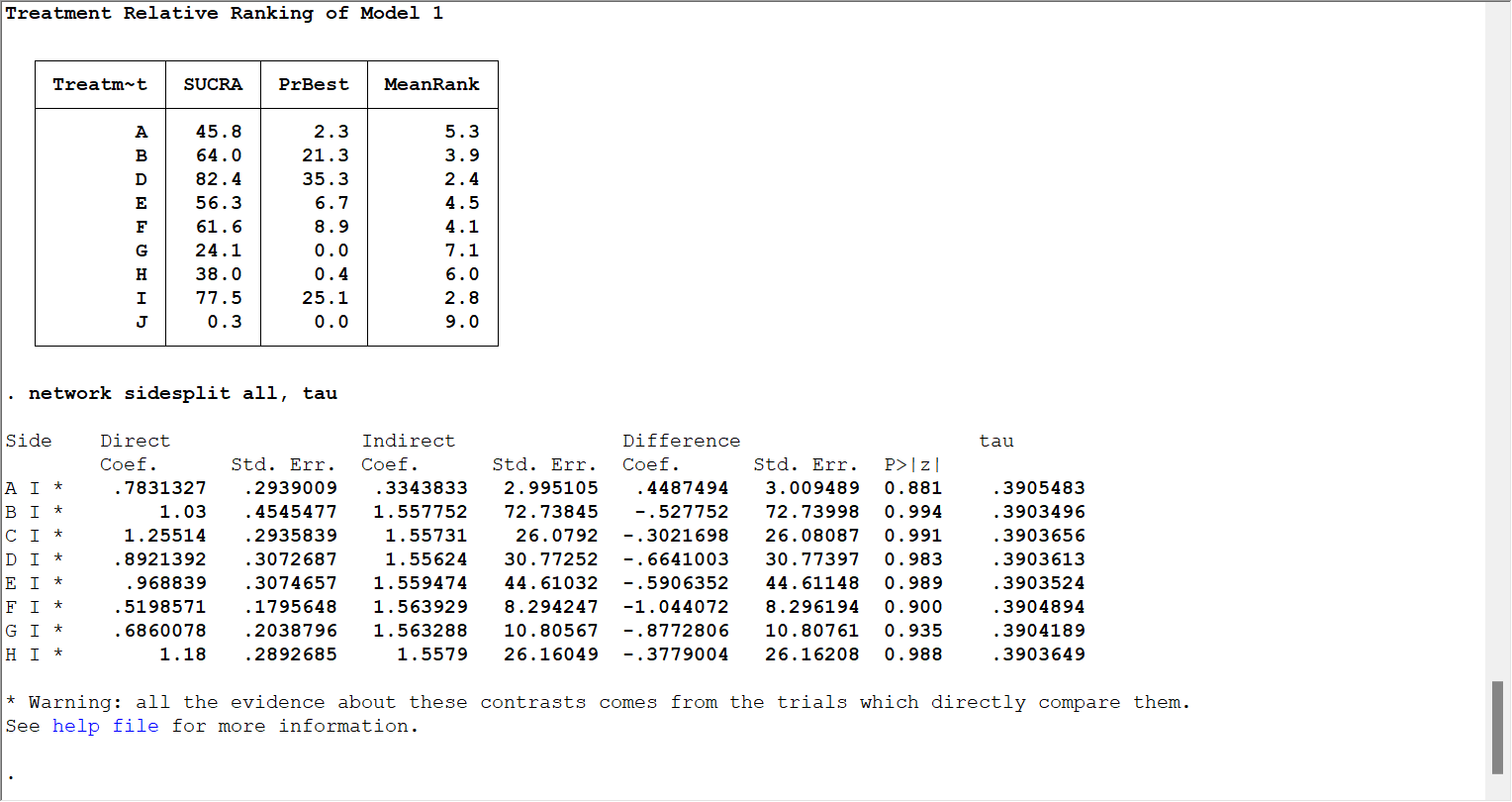


1. Nodal split analyses with whole blood reduced viscosity (high shear rate). A, Danhong injection+CT. B, Xuesaitong injection+CT. C, Dengzhanhuasu injection+CT. D, Shuxuening injection+CT. E, Guhong injection+CT. F, Shuxuetong injection+CT. G, Shenxiongputao injection+CT. H, Yinxingyetiquwu injection+CT. I, CT.


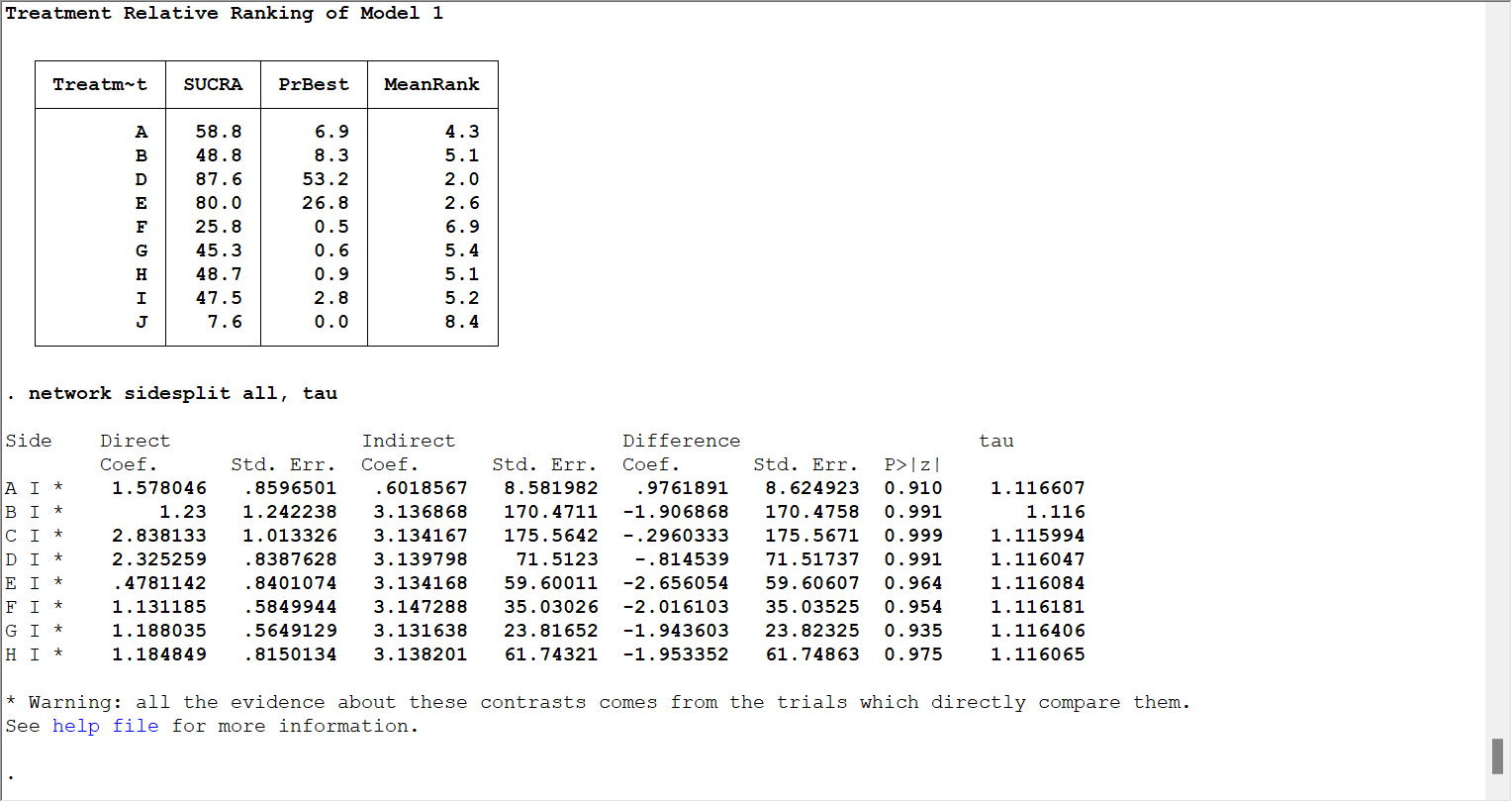


1. Nodal split analyses with whole blood reduced viscosity (low shear rate). A, Danhong injection+CT. B, Xuesaitong injection+CT. C, Dengzhanhuasu injection+CT. D, Shuxuening injection+CT. E, Guhong injection+CT. F, Shuxuetong injection+CT. G, Shenxiongputao injection+CT. H, Yinxingyetiquwu injection+CT. I, CT.


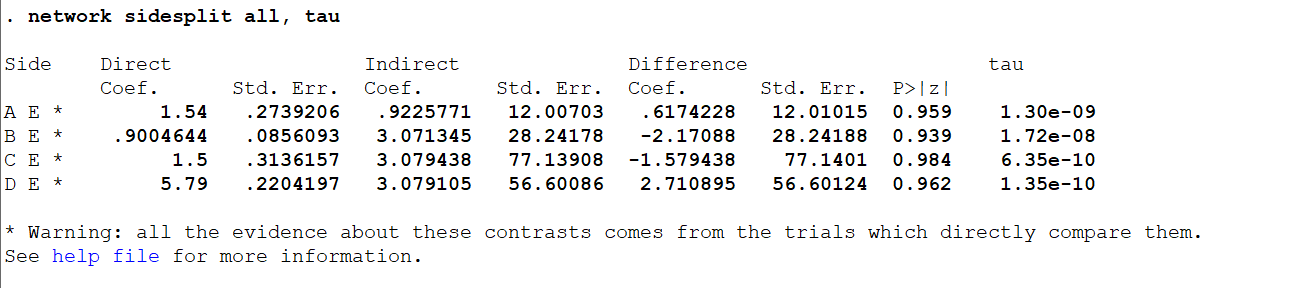


1. Nodal split analyses with TC. A, Danhong injection+CT. B, Xueshuantong injection+CT. C, Shuxuening injection+CT. D, Shuxuetong injection+CT. E, CT.


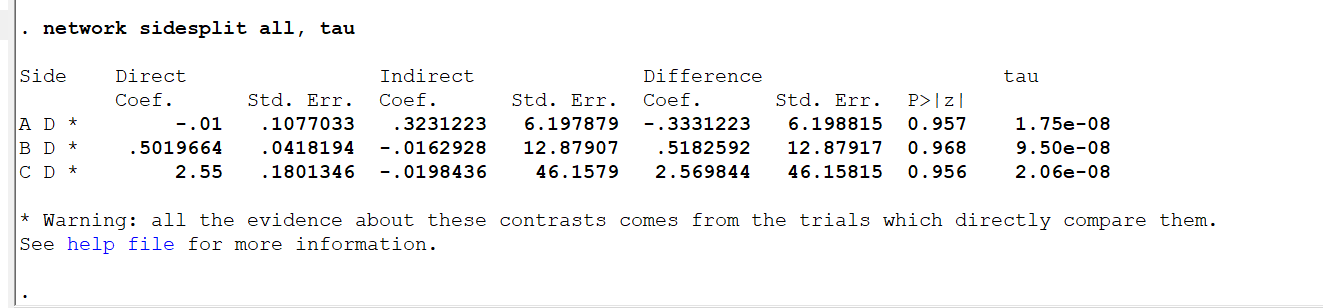


1. Nodal split analyses with TG. A, Danhong injection+CT. B, Xueshuantong injection+CT. C, Shuxuetong injection+CT. D, CT.


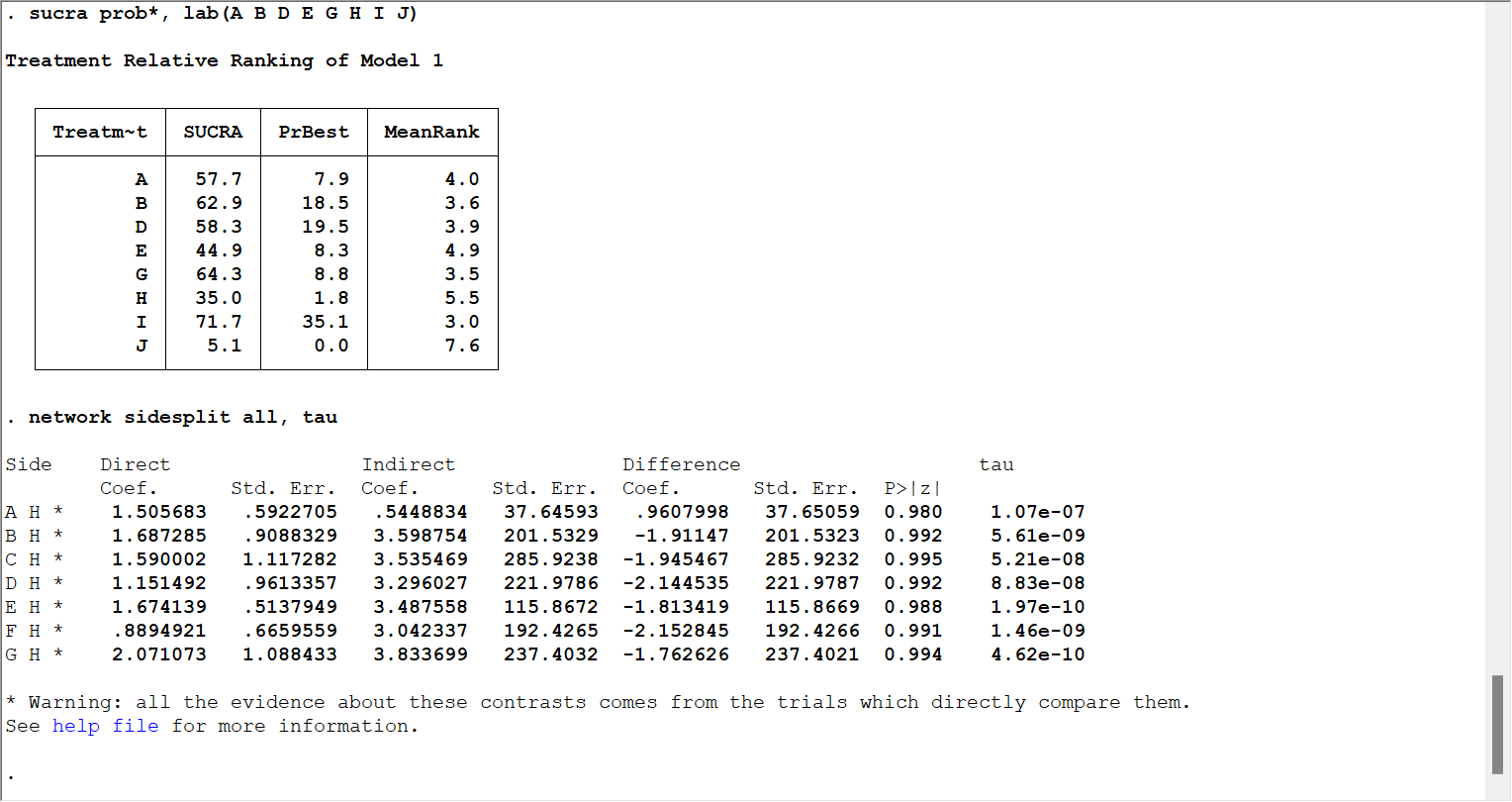


1. Nodal split analyses with Incidence of cerebral infarction. A, Danhong injection+CT. B, Xuesaitong injection+CT. C, Dengzhanhuasu injection+CT. D, Shuxuening injection+CT. E, Shuxuetong injection+CT. F, Shenxiongputao injection+CT. G, Yinxingyetiquwu injection+CT. H, CT.
