# Supplementary material for: Comparative efficacy and safety of traditional Chinese medicine injections in patients with transient ischemic attack: A systematic review and network meta-analysis
Source: PLoS One. 2024 Jul 24;19(7):e0307663. doi: 10.1371/journal.pone.0307663 (PMC11268667; doi:10.1371/journal.pone.0307663)
Supplement: S7 File — (DOCX) [file pone.0307663.s007.docx]

**S7 File. Grading the evidence for outcomes of the network meta-analysis using CINeMA.**

7.1 Total effectiveness rate

7.1.1 study limitations of the included studies


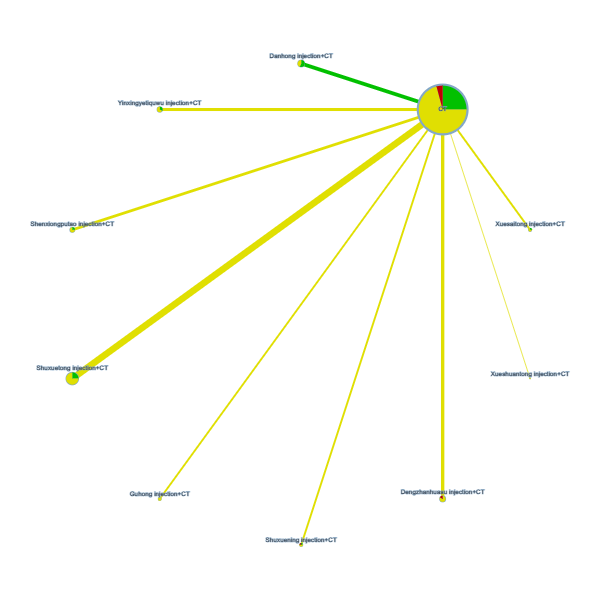


7.1.2 Contribution percentage of low, moderate, and high RoB comparisons to each network estimate

Low RoB is green, moderate RoB is yellow, high RoB is red.


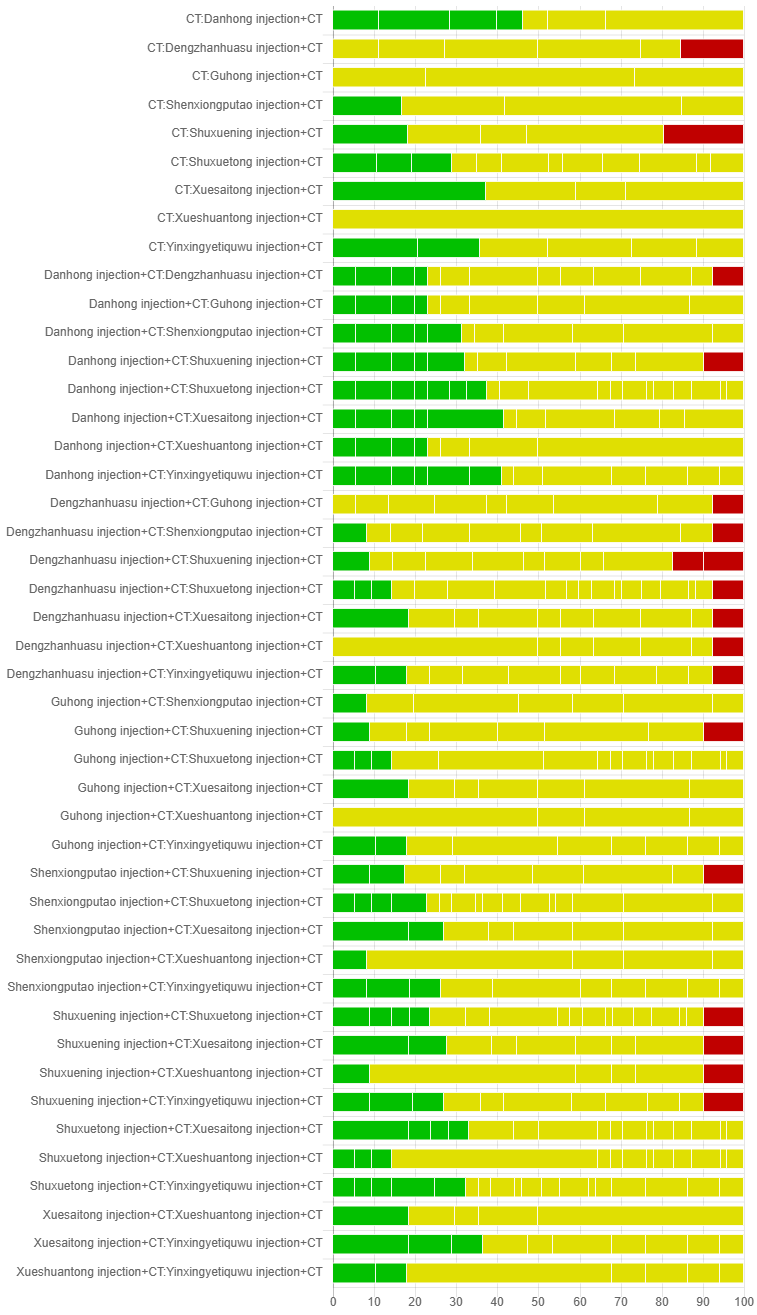


7.1.3 CINeMA for the primary outcome “total effectiveness rate”

| Comparison | Number of studies | Within-study bias | Reporting bias | Indirectness | Imprecision | Heterogeneity | Incoherence | Confidence rating |
| --- | --- | --- | --- | --- | --- | --- | --- | --- |
| CT:Danhong injection+CT | 7 | Some concerns | Low risk | No concerns | No concerns | No concerns | Major concerns | Very low |
| CT:Dengzhanhuasu injection+CT | 6 | Some concerns | Low risk | No concerns | No concerns | No concerns | Major concerns | Very low |
| CT:Guhong injection+CT | 3 | Some concerns | Low risk | No concerns | No concerns | No concerns | Major concerns | Very low |
| CT:Shenxiongputao injection+CT | 4 | Some concerns | Low risk | No concerns | No concerns | No concerns | Major concerns | Very low |
| CT:Shuxuening injection+CT | 5 | Some concerns | Low risk | No concerns | No concerns | No concerns | Major concerns | Very low |
| CT:Shuxuetong injection+CT | 12 | Some concerns | Low risk | No concerns | No concerns | No concerns | Major concerns | Very low |
| CT:Xuesaitong injection+CT | 4 | Some concerns | Low risk | No concerns | No concerns | No concerns | Major concerns | Very low |
| CT:Xueshuantong injection+CT | 1 | Some concerns | Low risk | No concerns | No concerns | No concerns | Major concerns | Very low |
| CT:Yinxingyetiquwu injection+CT | 6 | Some concerns | Low risk | No concerns | No concerns | No concerns | Major concerns | Very low |
| Danhong injection+CT:Dengzhanhuasu injection+CT | 0 | Some concerns | Low risk | No concerns | Major concerns | No concerns | Major concerns | Very low |
| Danhong injection+CT:Guhong injection+CT | 0 | Some concerns | Low risk | No concerns | Major concerns | No concerns | Major concerns | Very low |
| Danhong injection+CT:Shenxiongputao injection+CT | 0 | Some concerns | Low risk | No concerns | Major concerns | No concerns | Major concerns | Very low |
| Danhong injection+CT:Shuxuening injection+CT | 0 | Some concerns | Low risk | No concerns | Major concerns | No concerns | Major concerns | Very low |
| Danhong injection+CT:Shuxuetong injection+CT | 0 | Some concerns | Low risk | No concerns | Major concerns | No concerns | Major concerns | Very low |
| Danhong injection+CT:Xuesaitong injection+CT | 0 | Some concerns | Low risk | No concerns | Major concerns | No concerns | Major concerns | Very low |
| Danhong injection+CT:Xueshuantong injection+CT | 0 | Some concerns | Low risk | No concerns | Major concerns | No concerns | Major concerns | Very low |
| Danhong injection+CT:Yinxingyetiquwu injection+CT | 0 | Some concerns | Low risk | No concerns | Major concerns | No concerns | Major concerns | Very low |
| Dengzhanhuasu injection+CT:Guhong injection+CT | 0 | Some concerns | Low risk | No concerns | Major concerns | No concerns | Major concerns | Very low |
| Dengzhanhuasu injection+CT:Shenxiongputao injection+CT | 0 | Some concerns | Low risk | No concerns | Major concerns | No concerns | Major concerns | Very low |
| Dengzhanhuasu injection+CT:Shuxuening injection+CT | 0 | Some concerns | Low risk | No concerns | Major concerns | No concerns | Major concerns | Very low |
| Dengzhanhuasu injection+CT:Shuxuetong injection+CT | 0 | Some concerns | Low risk | No concerns | Major concerns | No concerns | Major concerns | Very low |
| Dengzhanhuasu injection+CT:Xuesaitong injection+CT | 0 | Some concerns | Low risk | No concerns | Major concerns | No concerns | Major concerns | Very low |
| Dengzhanhuasu injection+CT:Xueshuantong injection+CT | 0 | Some concerns | Low risk | No concerns | Major concerns | No concerns | Major concerns | Very low |
| Dengzhanhuasu injection+CT:Yinxingyetiquwu injection+CT | 0 | Some concerns | Low risk | No concerns | Major concerns | No concerns | Major concerns | Very low |
| Guhong injection+CT:Shenxiongputao injection+CT | 0 | Some concerns | Low risk | No concerns | Major concerns | No concerns | Major concerns | Very low |
| Guhong injection+CT:Shuxuening injection+CT | 0 | Some concerns | Low risk | No concerns | Major concerns | No concerns | Major concerns | Very low |
| Guhong injection+CT:Shuxuetong injection+CT | 0 | Some concerns | Low risk | No concerns | Major concerns | No concerns | Major concerns | Very low |
| Guhong injection+CT:Xuesaitong injection+CT | 0 | Some concerns | Low risk | No concerns | Major concerns | No concerns | Major concerns | Very low |
| Guhong injection+CT:Xueshuantong injection+CT | 0 | Some concerns | Low risk | No concerns | Major concerns | No concerns | Major concerns | Very low |
| Guhong injection+CT:Yinxingyetiquwu injection+CT | 0 | Some concerns | Low risk | No concerns | Major concerns | No concerns | Major concerns | Very low |
| Shenxiongputao injection+CT:Shuxuening injection+CT | 0 | Some concerns | Low risk | No concerns | Major concerns | No concerns | Major concerns | Very low |
| Shenxiongputao injection+CT:Shuxuetong injection+CT | 0 | Some concerns | Low risk | No concerns | Major concerns | No concerns | Major concerns | Very low |
| Shenxiongputao injection+CT:Xuesaitong injection+CT | 0 | Some concerns | Low risk | No concerns | Major concerns | No concerns | Major concerns | Very low |
| Shenxiongputao injection+CT:Xueshuantong injection+CT | 0 | Some concerns | Low risk | No concerns | Major concerns | No concerns | Major concerns | Very low |
| Shenxiongputao injection+CT:Yinxingyetiquwu injection+CT | 0 | Some concerns | Low risk | No concerns | Major concerns | No concerns | Major concerns | Very low |
| Shuxuening injection+CT:Shuxuetong injection+CT | 0 | Some concerns | Low risk | No concerns | Major concerns | No concerns | Major concerns | Very low |
| Shuxuening injection+CT:Xuesaitong injection+CT | 0 | Some concerns | Low risk | No concerns | Major concerns | No concerns | Major concerns | Very low |
| Shuxuening injection+CT:Xueshuantong injection+CT | 0 | Some concerns | Low risk | No concerns | Major concerns | No concerns | Major concerns | Very low |
| Shuxuening injection+CT:Yinxingyetiquwu injection+CT | 0 | Some concerns | Low risk | No concerns | Major concerns | No concerns | Major concerns | Very low |
| Shuxuetong injection+CT:Xuesaitong injection+CT | 0 | Some concerns | Low risk | No concerns | Major concerns | No concerns | Major concerns | Very low |
| Shuxuetong injection+CT:Xueshuantong injection+CT | 0 | Some concerns | Low risk | No concerns | Major concerns | No concerns | Major concerns | Very low |
| Shuxuetong injection+CT:Yinxingyetiquwu injection+CT | 0 | Some concerns | Low risk | No concerns | Major concerns | No concerns | Major concerns | Very low |
| Xuesaitong injection+CT:Xueshuantong injection+CT | 0 | Some concerns | Low risk | No concerns | Major concerns | No concerns | Major concerns | Very low |
| Xuesaitong injection+CT:Yinxingyetiquwu injection+CT | 0 | Some concerns | Low risk | No concerns | Major concerns | No concerns | Major concerns | Very low |
| Xueshuantong injection+CT:Yinxingyetiquwu injection+CT | 0 | Some concerns | Low risk | No concerns | Major concerns | No concerns | Major concerns | Very low |

7.2 Plasma viscosity

7.2.1 study limitations of the included studies


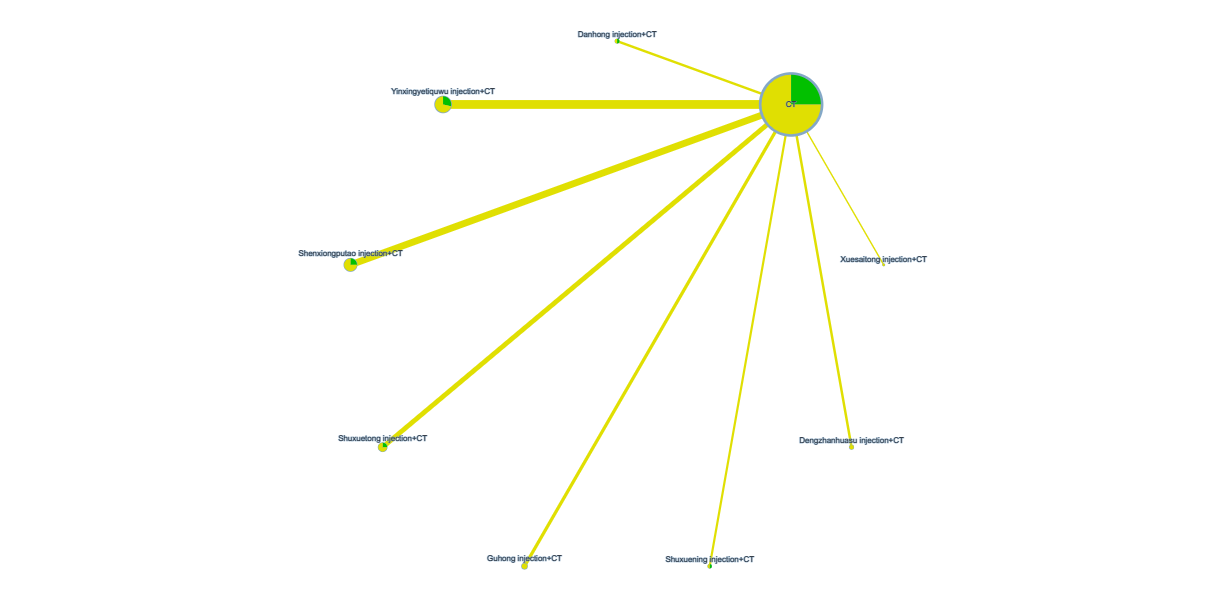


7.2.2 Contribution percentage of low, moderate, and high RoB comparisons to each network estimate

Low RoB is green, moderate RoB is yellow, high RoB is red.


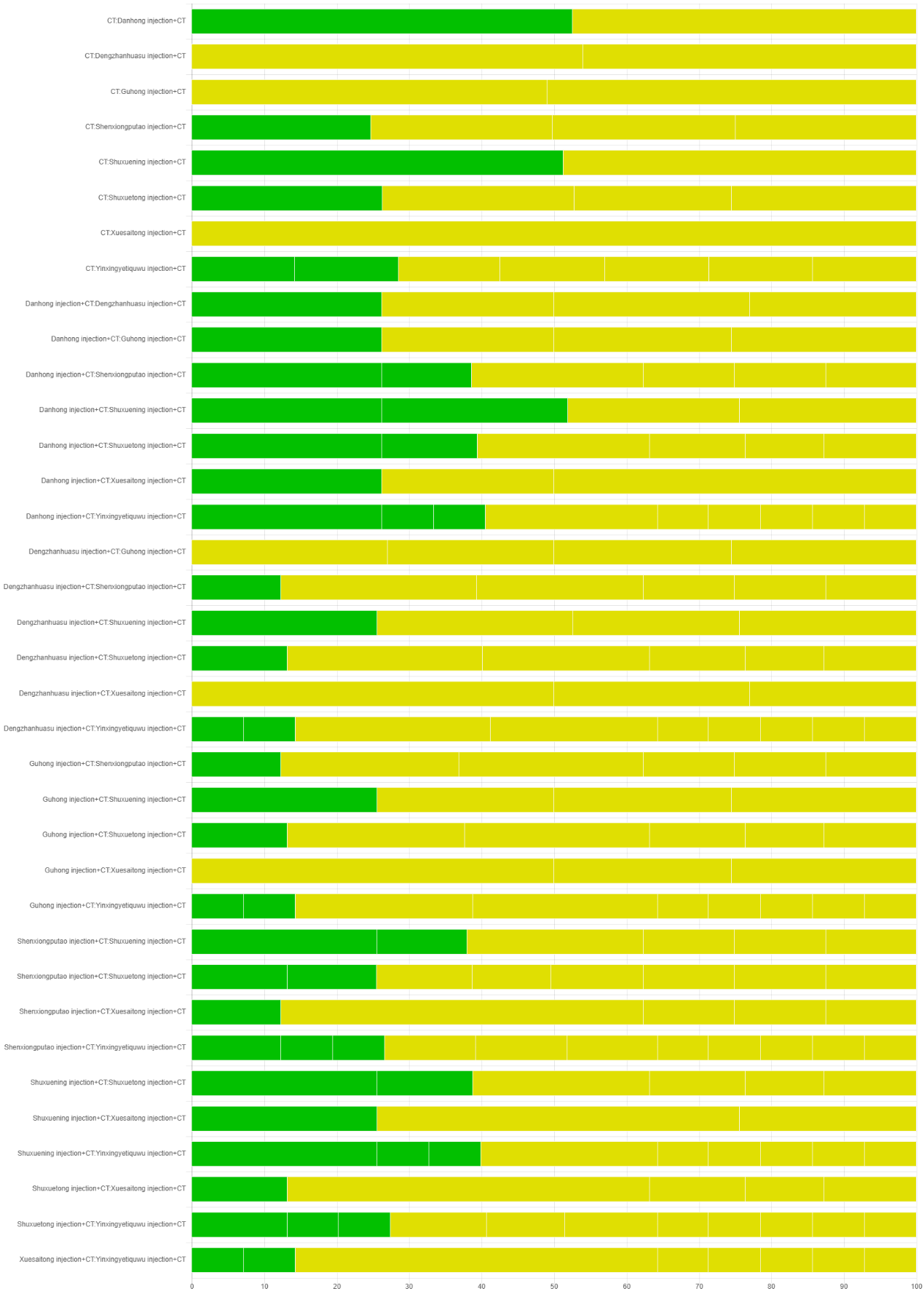


7.2.3 CINeMA for the primary outcome “plasma viscosity”

| Comparison | Number of studies | Within-study bias | Reporting bias | Indirectness | Imprecision | Heterogeneity | Incoherence | Confidence rating |
| --- | --- | --- | --- | --- | --- | --- | --- | --- |
| CT:Danhong injection+CT | 2 | No concerns | Low risk | No concerns | Major concerns | No concerns | Major concerns | Very low |
| CT:Dengzhanhuasu injection+CT | 2 | Some concerns | Low risk | No concerns | No concerns | No concerns | Major concerns | Very low |
| CT:Guhong injection+CT | 2 | Some concerns | Low risk | No concerns | No concerns | Major concerns | Major concerns | Very low |
| CT:Shenxiongputao injection+CT | 4 | Some concerns | Low risk | No concerns | No concerns | Major concerns | Major concerns | Very low |
| CT:Shuxuening injection+CT | 2 | No concerns | Low risk | No concerns | Major concerns | No concerns | Major concerns | Low |
| CT:Shuxuetong injection+CT | 4 | Some concerns | Low risk | No concerns | Major concerns | No concerns | Major concerns | Very low |
| CT:Xuesaitong injection+CT | 1 | Some concerns | Low risk | No concerns | Major concerns | No concerns | Major concerns | Very low |
| CT:Yinxingyetiquwu injection+CT | 7 | Some concerns | Low risk | No concerns | No concerns | Major concerns | Major concerns | Very low |
| Danhong injection+CT:Dengzhanhuasu injection+CT | 0 | Some concerns | Low risk | No concerns | No concerns | No concerns | Major concerns | Very low |
| Danhong injection+CT:Guhong injection+CT | 0 | Some concerns | Low risk | No concerns | Major concerns | No concerns | Major concerns | Very low |
| Danhong injection+CT:Shenxiongputao injection+CT | 0 | Some concerns | Low risk | No concerns | Major concerns | No concerns | Major concerns | Very low |
| Danhong injection+CT:Shuxuening injection+CT | 0 | No concerns | Low risk | No concerns | Major concerns | No concerns | Major concerns | Low |
| Danhong injection+CT:Shuxuetong injection+CT | 0 | Some concerns | Low risk | No concerns | Major concerns | No concerns | Major concerns | Very low |
| Danhong injection+CT:Xuesaitong injection+CT | 0 | Some concerns | Low risk | No concerns | Major concerns | No concerns | Major concerns | Very low |
| Danhong injection+CT:Yinxingyetiquwu injection+CT | 0 | Some concerns | Low risk | No concerns | Major concerns | No concerns | Major concerns | Very low |
| Dengzhanhuasu injection+CT:Guhong injection+CT | 0 | Some concerns | Low risk | No concerns | No concerns | No concerns | Major concerns | Very low |
| Dengzhanhuasu injection+CT:Shenxiongputao injection+CT | 0 | Some concerns | Low risk | No concerns | No concerns | No concerns | Major concerns | Very low |
| Dengzhanhuasu injection+CT:Shuxuening injection+CT | 0 | Some concerns | Low risk | No concerns | No concerns | No concerns | Major concerns | Very low |
| Dengzhanhuasu injection+CT:Shuxuetong injection+CT | 0 | Some concerns | Low risk | No concerns | No concerns | No concerns | Major concerns | Very low |
| Dengzhanhuasu injection+CT:Xuesaitong injection+CT | 0 | Some concerns | Low risk | No concerns | No concerns | No concerns | Major concerns | Very low |
| Dengzhanhuasu injection+CT:Yinxingyetiquwu injection+CT | 0 | Some concerns | Low risk | No concerns | No concerns | No concerns | Major concerns | Very low |
| Guhong injection+CT:Shenxiongputao injection+CT | 0 | Some concerns | Low risk | No concerns | Major concerns | No concerns | Major concerns | Very low |
| Guhong injection+CT:Shuxuening injection+CT | 0 | Some concerns | Low risk | No concerns | Major concerns | No concerns | Major concerns | Very low |
| Guhong injection+CT:Shuxuetong injection+CT | 0 | Some concerns | Low risk | No concerns | Major concerns | No concerns | Major concerns | Very low |
| Guhong injection+CT:Xuesaitong injection+CT | 0 | Some concerns | Low risk | No concerns | Major concerns | No concerns | Major concerns | Very low |
| Guhong injection+CT:Yinxingyetiquwu injection+CT | 0 | Some concerns | Low risk | No concerns | Major concerns | No concerns | Major concerns | Very low |
| Shenxiongputao injection+CT:Shuxuening injection+CT | 0 | Some concerns | Low risk | No concerns | Major concerns | No concerns | Major concerns | Very low |
| Shenxiongputao injection+CT:Shuxuetong injection+CT | 0 | Some concerns | Low risk | No concerns | Major concerns | No concerns | Major concerns | Very low |
| Shenxiongputao injection+CT:Xuesaitong injection+CT | 0 | Some concerns | Low risk | No concerns | Major concerns | No concerns | Major concerns | Very low |
| Shenxiongputao injection+CT:Yinxingyetiquwu injection+CT | 0 | Some concerns | Low risk | No concerns | Major concerns | No concerns | Major concerns | Very low |
| Shuxuening injection+CT:Shuxuetong injection+CT | 0 | Some concerns | Low risk | No concerns | Major concerns | No concerns | Major concerns | Very low |
| Shuxuening injection+CT:Xuesaitong injection+CT | 0 | Some concerns | Low risk | No concerns | Major concerns | No concerns | Major concerns | Very low |
| Shuxuening injection+CT:Yinxingyetiquwu injection+CT | 0 | Some concerns | Low risk | No concerns | Major concerns | No concerns | Major concerns | Very low |
| Shuxuetong injection+CT:Xuesaitong injection+CT | 0 | Some concerns | Low risk | No concerns | Major concerns | No concerns | Major concerns | Very low |
| Shuxuetong injection+CT:Yinxingyetiquwu injection+CT | 0 | Some concerns | Low risk | No concerns | Major concerns | No concerns | Major concerns | Very low |
| Xuesaitong injection+CT:Yinxingyetiquwu injection+CT | 0 | Some concerns | Low risk | No concerns | Major concerns | No concerns | Major concerns | Very low |

3 Fibrinogen

3.1 study limitations of the included studies


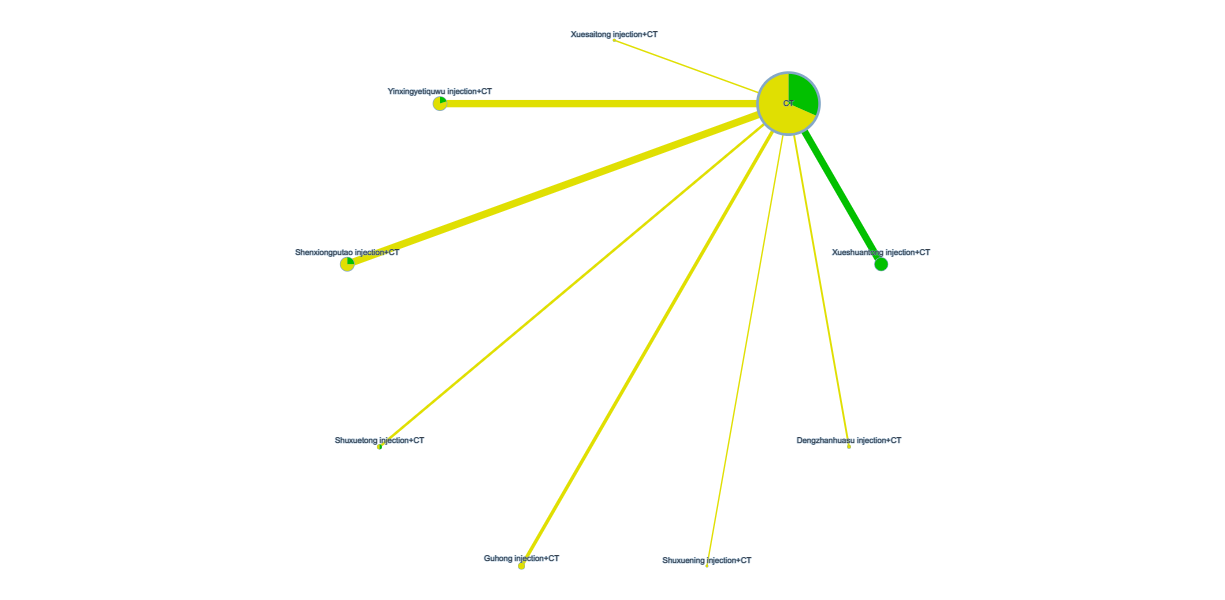


3.2 Contribution percentage of low, moderate, and high RoB comparisons to each network estimate

Low RoB is green, moderate RoB is yellow, high RoB is red.


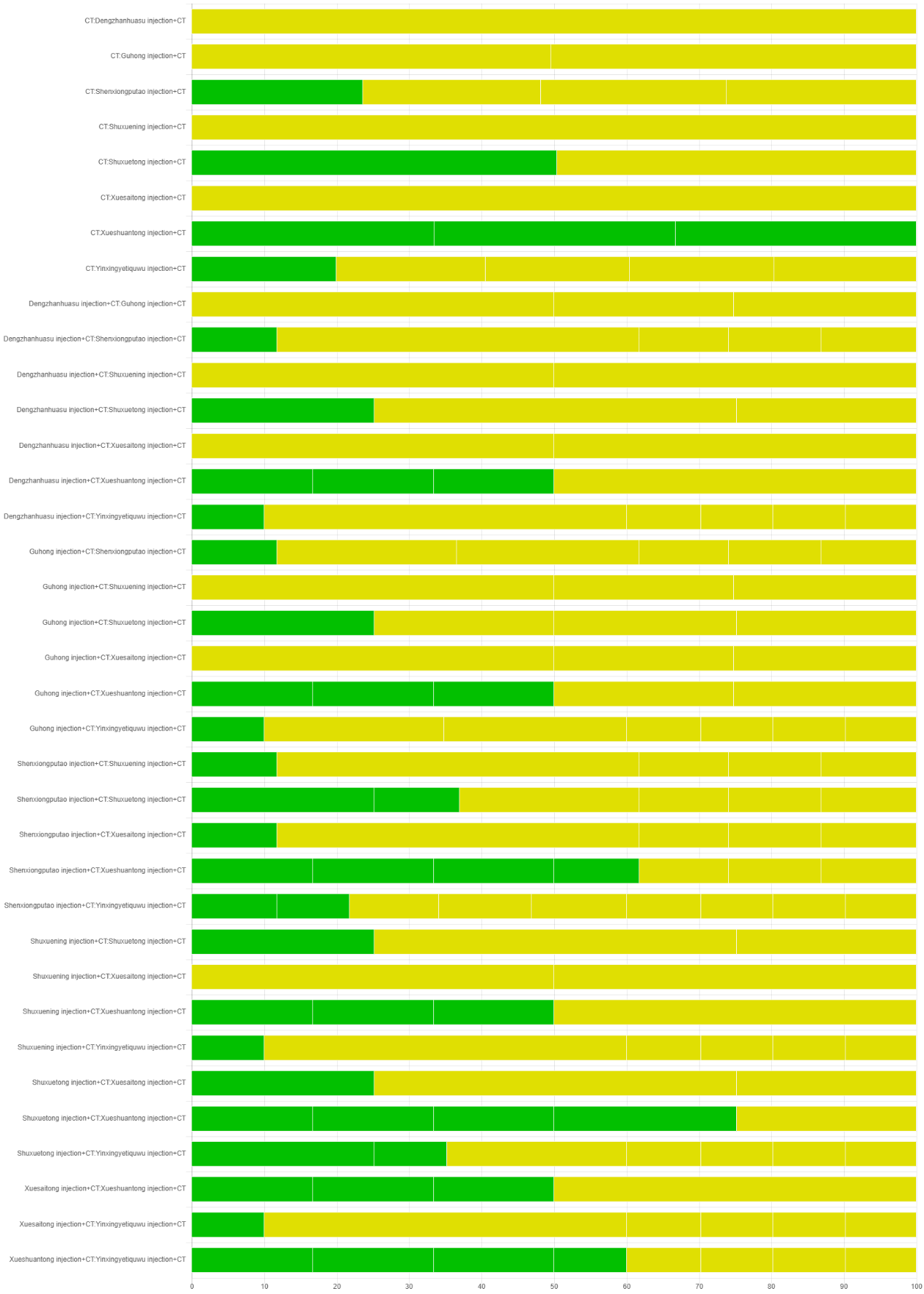


3.3 CINeMA for the primary outcome “fibrinogen”

| Comparison | Number of studies | Within-study bias | Reporting bias | Indirectness | Imprecision | Heterogeneity | Incoherence | Confidence rating |
| --- | --- | --- | --- | --- | --- | --- | --- | --- |
| CT:Dengzhanhuasu injection+CT | 1 | Some concerns | Low risk | No concerns | Major concerns | No concerns | Major concerns | Very low |
| CT:Guhong injection+CT | 2 | Some concerns | Low risk | No concerns | No concerns | Major concerns | Major concerns | Very low |
| CT:Shenxiongputao injection+CT | 4 | Some concerns | Low risk | No concerns | No concerns | Major concerns | Major concerns | Very low |
| CT:Shuxuening injection+CT | 1 | Some concerns | Low risk | No concerns | Major concerns | No concerns | Major concerns | Very low |
| CT:Shuxuetong injection+CT | 2 | No concerns | Low risk | No concerns | No concerns | No concerns | Major concerns | Low |
| CT:Xuesaitong injection+CT | 1 | Some concerns | Low risk | No concerns | Major concerns | No concerns | Major concerns | Very low |
| CT:Xueshuantong injection+CT | 3 | No concerns | Low risk | No concerns | Major concerns | No concerns | Major concerns | Low |
| CT:Yinxingyetiquwu injection+CT | 5 | Some concerns | Low risk | No concerns | No concerns | Major concerns | Major concerns | Very low |
| Dengzhanhuasu injection+CT:Guhong injection+CT | 0 | Some concerns | Low risk | No concerns | Major concerns | No concerns | Major concerns | Very low |
| Dengzhanhuasu injection+CT:Shenxiongputao injection+CT | 0 | Some concerns | Low risk | No concerns | Major concerns | No concerns | Major concerns | Very low |
| Dengzhanhuasu injection+CT:Shuxuening injection+CT | 0 | Some concerns | Low risk | No concerns | Major concerns | No concerns | Major concerns | Very low |
| Dengzhanhuasu injection+CT:Shuxuetong injection+CT | 0 | Some concerns | Low risk | No concerns | Major concerns | No concerns | Major concerns | Very low |
| Dengzhanhuasu injection+CT:Xuesaitong injection+CT | 0 | Some concerns | Low risk | No concerns | Major concerns | No concerns | Major concerns | Very low |
| Dengzhanhuasu injection+CT:Xueshuantong injection+CT | 0 | No concerns | Low risk | No concerns | Major concerns | No concerns | Major concerns | Low |
| Dengzhanhuasu injection+CT:Yinxingyetiquwu injection+CT | 0 | Some concerns | Low risk | No concerns | Major concerns | No concerns | Major concerns | Very low |
| Guhong injection+CT:Shenxiongputao injection+CT | 0 | Some concerns | Low risk | No concerns | Major concerns | No concerns | Major concerns | Very low |
| Guhong injection+CT:Shuxuening injection+CT | 0 | Some concerns | Low risk | No concerns | Major concerns | No concerns | Major concerns | Very low |
| Guhong injection+CT:Shuxuetong injection+CT | 0 | Some concerns | Low risk | No concerns | Major concerns | No concerns | Major concerns | Very low |
| Guhong injection+CT:Xuesaitong injection+CT | 0 | Some concerns | Low risk | No concerns | Major concerns | No concerns | Major concerns | Very low |
| Guhong injection+CT:Xueshuantong injection+CT | 0 | No concerns | Low risk | No concerns | Major concerns | No concerns | Major concerns | Low |
| Guhong injection+CT:Yinxingyetiquwu injection+CT | 0 | Some concerns | Low risk | No concerns | Major concerns | No concerns | Major concerns | Very low |
| Shenxiongputao injection+CT:Shuxuening injection+CT | 0 | Some concerns | Low risk | No concerns | Major concerns | No concerns | Major concerns | Very low |
| Shenxiongputao injection+CT:Shuxuetong injection+CT | 0 | Some concerns | Low risk | No concerns | Major concerns | No concerns | Major concerns | Very low |
| Shenxiongputao injection+CT:Xuesaitong injection+CT | 0 | Some concerns | Low risk | No concerns | Major concerns | No concerns | Major concerns | Very low |
| Shenxiongputao injection+CT:Xueshuantong injection+CT | 0 | No concerns | Low risk | No concerns | Major concerns | No concerns | Major concerns | Low |
| Shenxiongputao injection+CT:Yinxingyetiquwu injection+CT | 0 | Some concerns | Low risk | No concerns | Major concerns | No concerns | Major concerns | Very low |
| Shuxuening injection+CT:Shuxuetong injection+CT | 0 | Some concerns | Low risk | No concerns | Major concerns | No concerns | Major concerns | Very low |
| Shuxuening injection+CT:Xuesaitong injection+CT | 0 | Some concerns | Low risk | No concerns | Major concerns | No concerns | Major concerns | Very low |
| Shuxuening injection+CT:Xueshuantong injection+CT | 0 | No concerns | Low risk | No concerns | Major concerns | No concerns | Major concerns | Low |
| Shuxuening injection+CT:Yinxingyetiquwu injection+CT | 0 | Some concerns | Low risk | No concerns | Major concerns | No concerns | Major concerns | Very low |
| Shuxuetong injection+CT:Xuesaitong injection+CT | 0 | Some concerns | Low risk | No concerns | Major concerns | No concerns | Major concerns | Very low |
| Shuxuetong injection+CT:Xueshuantong injection+CT | 0 | No concerns | Low risk | No concerns | No concerns | Major concerns | Major concerns | Low |
| Shuxuetong injection+CT:Yinxingyetiquwu injection+CT | 0 | Some concerns | Low risk | No concerns | Major concerns | No concerns | Major concerns | Very low |
| Xuesaitong injection+CT:Xueshuantong injection+CT | 0 | No concerns | Low risk | No concerns | Major concerns | No concerns | Major concerns | Low |
| Xuesaitong injection+CT:Yinxingyetiquwu injection+CT | 0 | Some concerns | Low risk | No concerns | Major concerns | No concerns | Major concerns | Very low |
| Xueshuantong injection+CT:Yinxingyetiquwu injection+CT | 0 | No concerns | Low risk | No concerns | Major concerns | No concerns | Major concerns | Low |

4 Whole blood reduced viscosity (high shear rate)

4.1 study limitations of the included studies


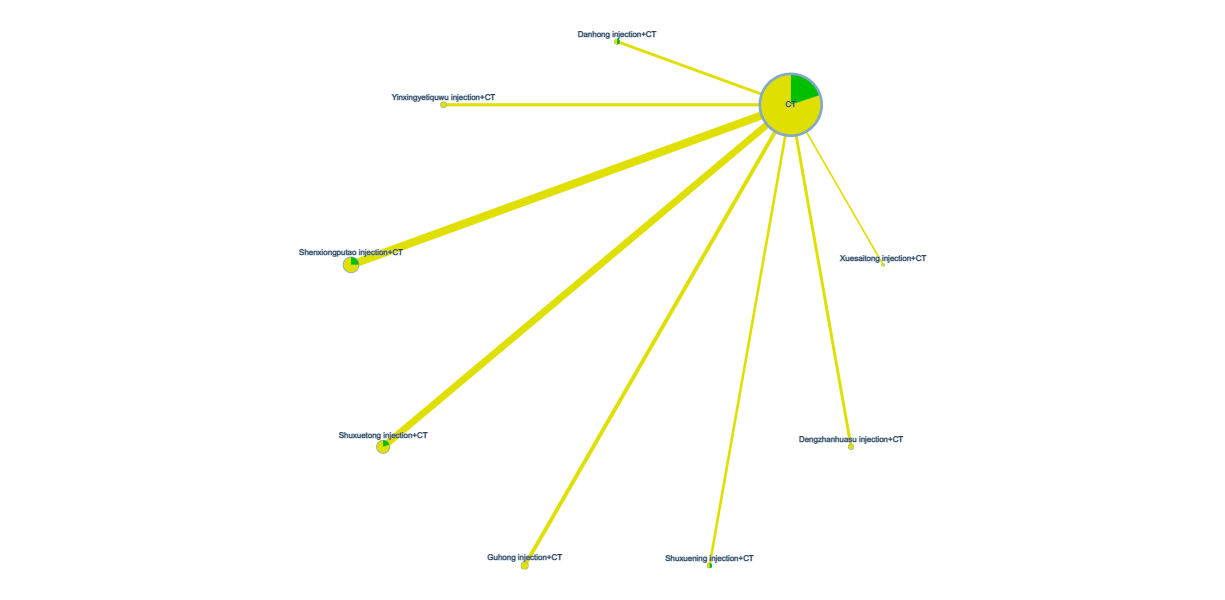


4.2 Contribution percentage of low, moderate, and high RoB comparisons to each network estimate

Low RoB is green, moderate RoB is yellow, high RoB is red.


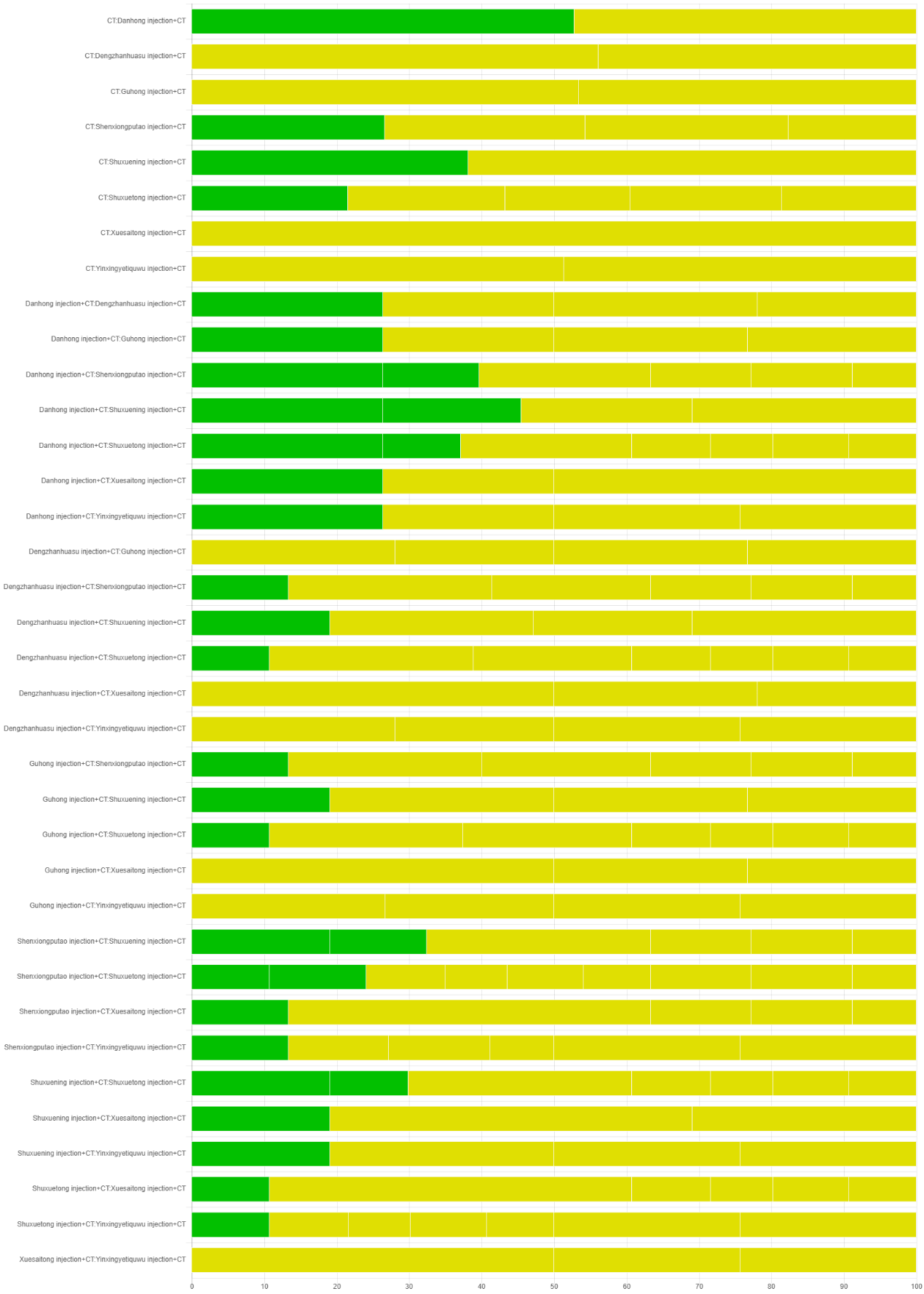


4.3 CINeMA for the primary outcome “Whole blood reduced viscosity (high shear rate)”

| Comparison | Number of studies | Within-study bias | Reporting bias | Indirectness | Imprecision | Heterogeneity | Incoherence | Confidence rating |
| --- | --- | --- | --- | --- | --- | --- | --- | --- |
| CT:Danhong injection+CT | 2 | No concerns | Low risk | No concerns | No concerns | No concerns | Major concerns | Low |
| CT:Dengzhanhuasu injection+CT | 2 | Some concerns | Low risk | No concerns | No concerns | No concerns | Major concerns | Very low |
| CT:Guhong injection+CT | 2 | Some concerns | Low risk | No concerns | No concerns | No concerns | Major concerns | Very low |
| CT:Shenxiongputao injection+CT | 4 | Some concerns | Low risk | No concerns | No concerns | Major concerns | Major concerns | Very low |
| CT:Shuxuening injection+CT | 2 | Some concerns | Low risk | No concerns | No concerns | No concerns | Major concerns | Very low |
| CT:Shuxuetong injection+CT | 5 | Some concerns | Low risk | No concerns | No concerns | Major concerns | Major concerns | Very low |
| CT:Xuesaitong injection+CT | 1 | Some concerns | Low risk | No concerns | No concerns | No concerns | Major concerns | Very low |
| CT:Yinxingyetiquwu injection+CT | 2 | Some concerns | Low risk | No concerns | No concerns | No concerns | Major concerns | Very low |
| Danhong injection+CT:Dengzhanhuasu injection+CT | 0 | Some concerns | Low risk | No concerns | Major concerns | No concerns | Major concerns | Very low |
| Danhong injection+CT:Guhong injection+CT | 0 | Some concerns | Low risk | No concerns | Major concerns | No concerns | Major concerns | Very low |
| Danhong injection+CT:Shenxiongputao injection+CT | 0 | Some concerns | Low risk | No concerns | Major concerns | No concerns | Major concerns | Very low |
| Danhong injection+CT:Shuxuening injection+CT | 0 | Some concerns | Low risk | No concerns | Major concerns | No concerns | Major concerns | Very low |
| Danhong injection+CT:Shuxuetong injection+CT | 0 | Some concerns | Low risk | No concerns | Major concerns | No concerns | Major concerns | Very low |
| Danhong injection+CT:Xuesaitong injection+CT | 0 | Some concerns | Low risk | No concerns | Major concerns | No concerns | Major concerns | Very low |
| Danhong injection+CT:Yinxingyetiquwu injection+CT | 0 | Some concerns | Low risk | No concerns | Major concerns | No concerns | Major concerns | Very low |
| Dengzhanhuasu injection+CT:Guhong injection+CT | 0 | Some concerns | Low risk | No concerns | Major concerns | No concerns | Major concerns | Very low |
| Dengzhanhuasu injection+CT:Shenxiongputao injection+CT | 0 | Some concerns | Low risk | No concerns | No concerns | Major concerns | Major concerns | Very low |
| Dengzhanhuasu injection+CT:Shuxuening injection+CT | 0 | Some concerns | Low risk | No concerns | Major concerns | No concerns | Major concerns | Very low |
| Dengzhanhuasu injection+CT:Shuxuetong injection+CT | 0 | Some concerns | Low risk | No concerns | No concerns | Major concerns | Major concerns | Very low |
| Dengzhanhuasu injection+CT:Xuesaitong injection+CT | 0 | Some concerns | Low risk | No concerns | Major concerns | No concerns | Major concerns | Very low |
| Dengzhanhuasu injection+CT:Yinxingyetiquwu injection+CT | 0 | Some concerns | Low risk | No concerns | Major concerns | No concerns | Major concerns | Very low |
| Guhong injection+CT:Shenxiongputao injection+CT | 0 | Some concerns | Low risk | No concerns | Major concerns | No concerns | Major concerns | Very low |
| Guhong injection+CT:Shuxuening injection+CT | 0 | Some concerns | Low risk | No concerns | Major concerns | No concerns | Major concerns | Very low |
| Guhong injection+CT:Shuxuetong injection+CT | 0 | Some concerns | Low risk | No concerns | Major concerns | No concerns | Major concerns | Very low |
| Guhong injection+CT:Xuesaitong injection+CT | 0 | Some concerns | Low risk | No concerns | Major concerns | No concerns | Major concerns | Very low |
| Guhong injection+CT:Yinxingyetiquwu injection+CT | 0 | Some concerns | Low risk | No concerns | Major concerns | No concerns | Major concerns | Very low |
| Shenxiongputao injection+CT:Shuxuening injection+CT | 0 | Some concerns | Low risk | No concerns | Major concerns | No concerns | Major concerns | Very low |
| Shenxiongputao injection+CT:Shuxuetong injection+CT | 0 | Some concerns | Low risk | No concerns | Major concerns | No concerns | Major concerns | Very low |
| Shenxiongputao injection+CT:Xuesaitong injection+CT | 0 | Some concerns | Low risk | No concerns | Major concerns | No concerns | Major concerns | Very low |
| Shenxiongputao injection+CT:Yinxingyetiquwu injection+CT | 0 | Some concerns | Low risk | No concerns | No concerns | Major concerns | Major concerns | Very low |
| Shuxuening injection+CT:Shuxuetong injection+CT | 0 | Some concerns | Low risk | No concerns | Major concerns | No concerns | Major concerns | Very low |
| Shuxuening injection+CT:Xuesaitong injection+CT | 0 | Some concerns | Low risk | No concerns | Major concerns | No concerns | Major concerns | Very low |
| Shuxuening injection+CT:Yinxingyetiquwu injection+CT | 0 | Some concerns | Low risk | No concerns | Major concerns | No concerns | Major concerns | Very low |
| Shuxuetong injection+CT:Xuesaitong injection+CT | 0 | Some concerns | Low risk | No concerns | Major concerns | No concerns | Major concerns | Very low |
| Shuxuetong injection+CT:Yinxingyetiquwu injection+CT | 0 | Some concerns | Low risk | No concerns | No concerns | Major concerns | Major concerns | Very low |
| Xuesaitong injection+CT:Yinxingyetiquwu injection+CT | 0 | Some concerns | Low risk | No concerns | Major concerns | No concerns | Major concerns | Very low |

5 Whole blood reduced viscosity (low shear rate)

5.1 study limitations of the included studies


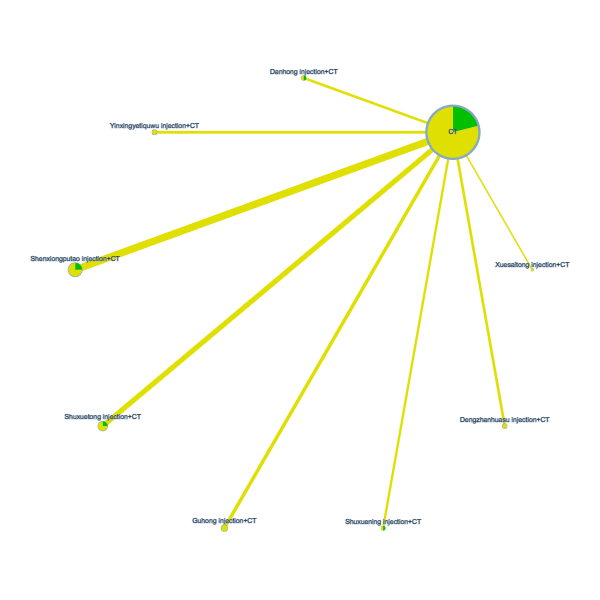


5.2 Contribution percentage of low, moderate, and high RoB comparisons to each network estimate

Low RoB is green, moderate RoB is yellow, high RoB is red.


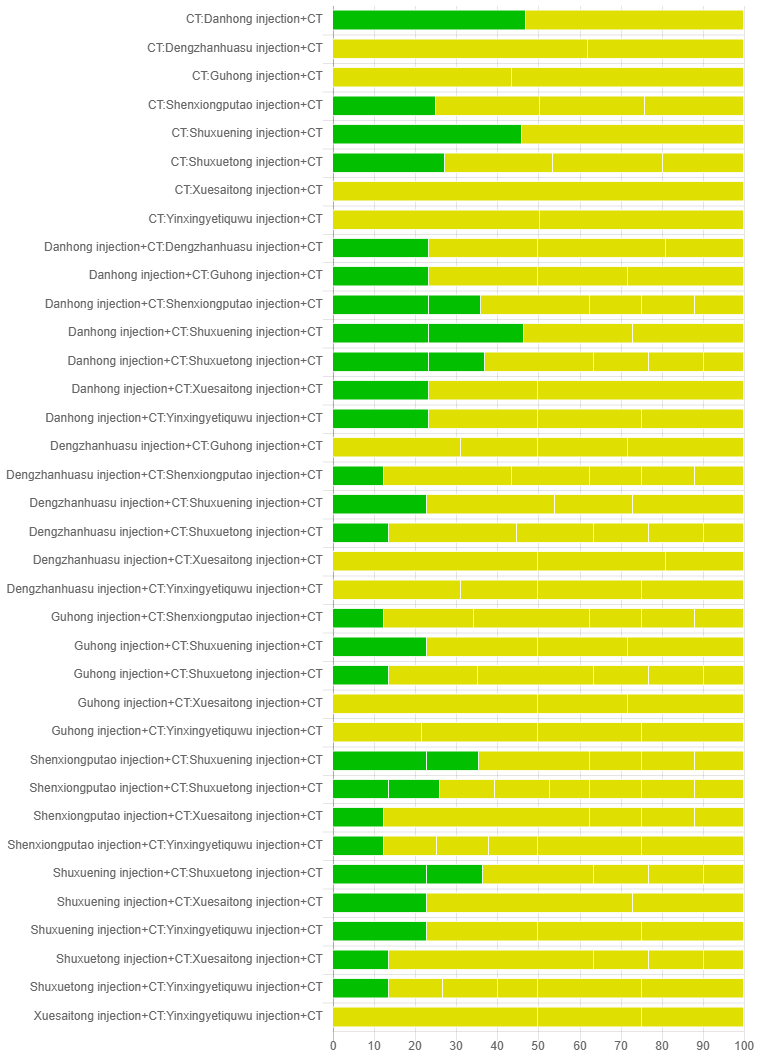


5.3 CINeMA for the primary outcome “Whole blood reduced viscosity (low shear rate)”

| Comparison | Number of studies | Within-study bias | Reporting bias | Indirectness | Imprecision | Heterogeneity | Incoherence | Confidence rating |
| --- | --- | --- | --- | --- | --- | --- | --- | --- |
| CT:Danhong injection+CT | 2 | Some concerns | Low risk | No concerns | No concerns | Major concerns | Major concerns | Very low |
| CT:Dengzhanhuasu injection+CT | 2 | Some concerns | Low risk | No concerns | No concerns | Major concerns | Major concerns | Very low |
| CT:Guhong injection+CT | 2 | Some concerns | Low risk | No concerns | Major concerns | No concerns | Major concerns | Very low |
| CT:Shenxiongputao injection+CT | 4 | Some concerns | Low risk | No concerns | No concerns | Major concerns | Major concerns | Very low |
| CT:Shuxuening injection+CT | 2 | Some concerns | Low risk | No concerns | No concerns | Major concerns | Major concerns | Very low |
| CT:Shuxuetong injection+CT | 4 | Some concerns | Low risk | No concerns | No concerns | Major concerns | Major concerns | Very low |
| CT:Xuesaitong injection+CT | 1 | Some concerns | Low risk | No concerns | Major concerns | No concerns | Major concerns | Very low |
| CT:Yinxingyetiquwu injection+CT | 2 | Some concerns | Low risk | No concerns | Major concerns | No concerns | Major concerns | Very low |
| Danhong injection+CT:Dengzhanhuasu injection+CT | 0 | Some concerns | Low risk | No concerns | Major concerns | No concerns | Major concerns | Very low |
| Danhong injection+CT:Guhong injection+CT | 0 | Some concerns | Low risk | No concerns | Major concerns | No concerns | Major concerns | Very low |
| Danhong injection+CT:Shenxiongputao injection+CT | 0 | Some concerns | Low risk | No concerns | Major concerns | No concerns | Major concerns | Very low |
| Danhong injection+CT:Shuxuening injection+CT | 0 | Some concerns | Low risk | No concerns | Major concerns | No concerns | Major concerns | Very low |
| Danhong injection+CT:Shuxuetong injection+CT | 0 | Some concerns | Low risk | No concerns | Major concerns | No concerns | Major concerns | Very low |
| Danhong injection+CT:Xuesaitong injection+CT | 0 | Some concerns | Low risk | No concerns | Major concerns | No concerns | Major concerns | Very low |
| Danhong injection+CT:Yinxingyetiquwu injection+CT | 0 | Some concerns | Low risk | No concerns | Major concerns | No concerns | Major concerns | Very low |
| Dengzhanhuasu injection+CT:Guhong injection+CT | 0 | Some concerns | Low risk | No concerns | No concerns | Major concerns | Major concerns | Very low |
| Dengzhanhuasu injection+CT:Shenxiongputao injection+CT | 0 | Some concerns | Low risk | No concerns | Major concerns | No concerns | Major concerns | Very low |
| Dengzhanhuasu injection+CT:Shuxuening injection+CT | 0 | Some concerns | Low risk | No concerns | Major concerns | No concerns | Major concerns | Very low |
| Dengzhanhuasu injection+CT:Shuxuetong injection+CT | 0 | Some concerns | Low risk | No concerns | Major concerns | No concerns | Major concerns | Very low |
| Dengzhanhuasu injection+CT:Xuesaitong injection+CT | 0 | Some concerns | Low risk | No concerns | Major concerns | No concerns | Major concerns | Very low |
| Dengzhanhuasu injection+CT:Yinxingyetiquwu injection+CT | 0 | Some concerns | Low risk | No concerns | Major concerns | No concerns | Major concerns | Very low |
| Guhong injection+CT:Shenxiongputao injection+CT | 0 | Some concerns | Low risk | No concerns | Major concerns | No concerns | Major concerns | Very low |
| Guhong injection+CT:Shuxuening injection+CT | 0 | Some concerns | Low risk | No concerns | Major concerns | No concerns | Major concerns | Very low |
| Guhong injection+CT:Shuxuetong injection+CT | 0 | Some concerns | Low risk | No concerns | Major concerns | No concerns | Major concerns | Very low |
| Guhong injection+CT:Xuesaitong injection+CT | 0 | Some concerns | Low risk | No concerns | Major concerns | No concerns | Major concerns | Very low |
| Guhong injection+CT:Yinxingyetiquwu injection+CT | 0 | Some concerns | Low risk | No concerns | Major concerns | No concerns | Major concerns | Very low |
| Shenxiongputao injection+CT:Shuxuening injection+CT | 0 | Some concerns | Low risk | No concerns | Major concerns | No concerns | Major concerns | Very low |
| Shenxiongputao injection+CT:Shuxuetong injection+CT | 0 | Some concerns | Low risk | No concerns | Major concerns | No concerns | Major concerns | Very low |
| Shenxiongputao injection+CT:Xuesaitong injection+CT | 0 | Some concerns | Low risk | No concerns | Major concerns | No concerns | Major concerns | Very low |
| Shenxiongputao injection+CT:Yinxingyetiquwu injection+CT | 0 | Some concerns | Low risk | No concerns | Major concerns | No concerns | Major concerns | Very low |
| Shuxuening injection+CT:Shuxuetong injection+CT | 0 | Some concerns | Low risk | No concerns | Major concerns | No concerns | Major concerns | Very low |
| Shuxuening injection+CT:Xuesaitong injection+CT | 0 | Some concerns | Low risk | No concerns | Major concerns | No concerns | Major concerns | Very low |
| Shuxuening injection+CT:Yinxingyetiquwu injection+CT | 0 | Some concerns | Low risk | No concerns | Major concerns | No concerns | Major concerns | Very low |
| Shuxuetong injection+CT:Xuesaitong injection+CT | 0 | Some concerns | Low risk | No concerns | Major concerns | No concerns | Major concerns | Very low |
| Shuxuetong injection+CT:Yinxingyetiquwu injection+CT | 0 | Some concerns | Low risk | No concerns | Major concerns | No concerns | Major concerns | Very low |
| Xuesaitong injection+CT:Yinxingyetiquwu injection+CT | 0 | Some concerns | Low risk | No concerns | Major concerns | No concerns | Major concerns | Very low |

6 Total cholesterol

6.1 study limitations of the included studies


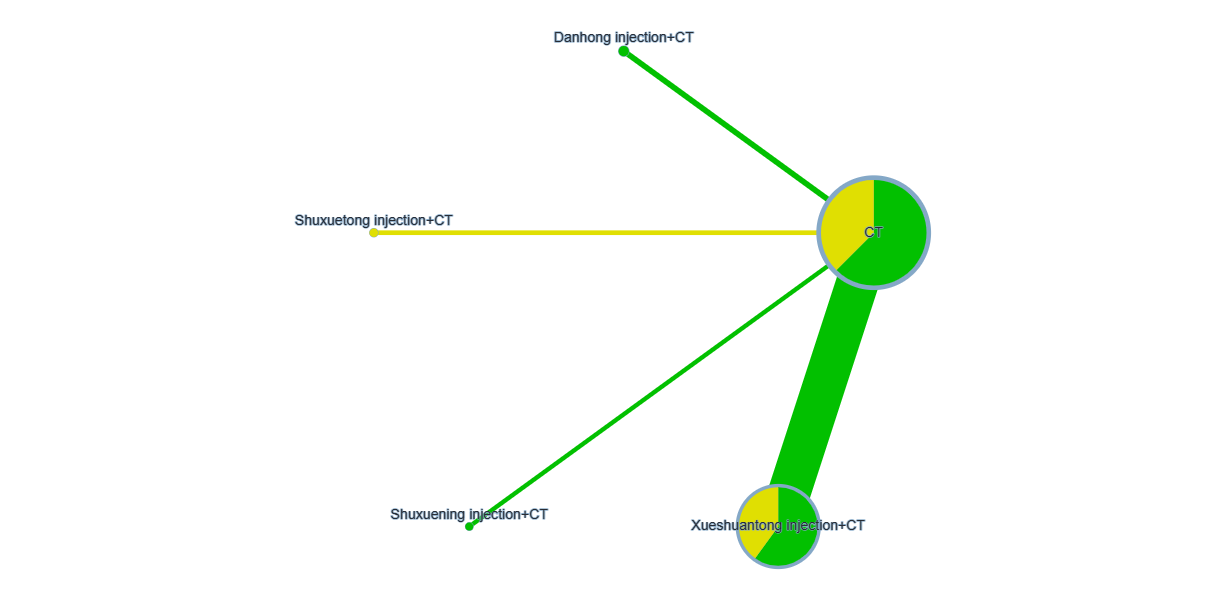


6.2 Contribution percentage of low, moderate, and high RoB comparisons to each network estimate

Low RoB is green, moderate RoB is yellow, high RoB is red.


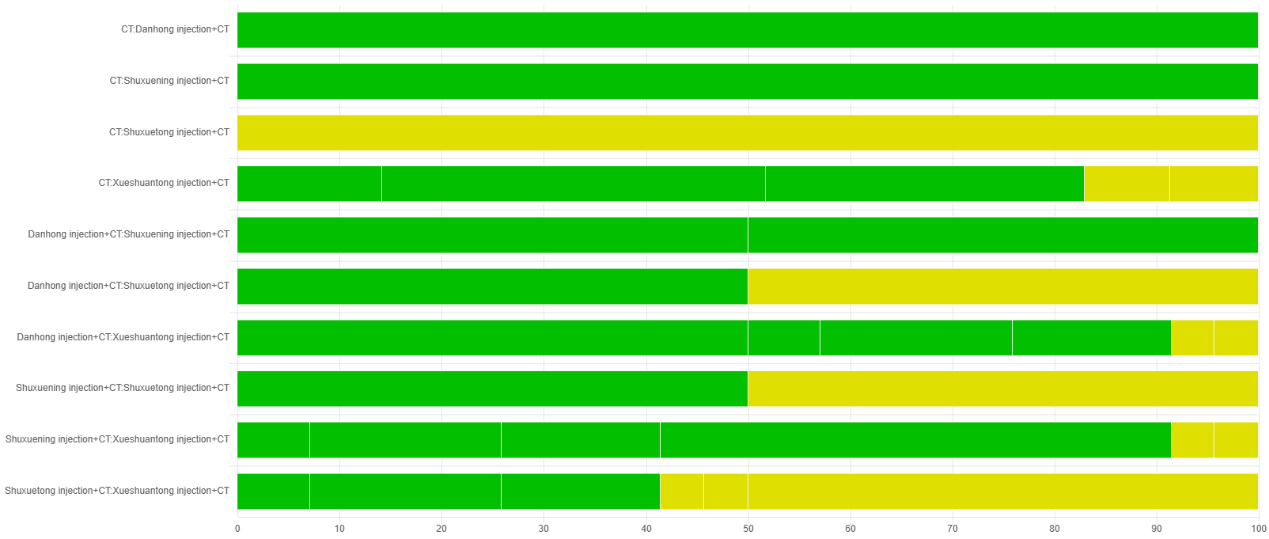


6.3 CINeMA for the primary outcome “total cholesterol”

| Comparison | Number of studies | Within-study bias | Reporting bias | Indirectness | Imprecision | Heterogeneity | Incoherence | Confidence rating |
| --- | --- | --- | --- | --- | --- | --- | --- | --- |
| CT:Danhong injection+CT | 1 | No concerns | Low risk | No concerns | No concerns | No concerns | Major concerns | Low |
| CT:Shuxuening injection+CT | 1 | No concerns | Low risk | No concerns | No concerns | No concerns | Major concerns | Low |
| CT:Shuxuetong injection+CT | 1 | Some concerns | Low risk | No concerns | No concerns | No concerns | Major concerns | Very low |
| CT:Xueshuantong injection+CT | 5 | No concerns | Low risk | No concerns | No concerns | No concerns | Major concerns | Low |
| Danhong injection+CT:Shuxuening injection+CT | 0 | No concerns | Low risk | No concerns | Major concerns | No concerns | Major concerns | Low |
| Danhong injection+CT:Shuxuetong injection+CT | 0 | Some concerns | Low risk | No concerns | No concerns | No concerns | Major concerns | Very low |
| Danhong injection+CT:Xueshuantong injection+CT | 0 | No concerns | Low risk | No concerns | No concerns | Major concerns | Major concerns | Low |
| Shuxuening injection+CT:Shuxuetong injection+CT | 0 | Some concerns | Low risk | No concerns | No concerns | No concerns | Major concerns | Very low |
| Shuxuening injection+CT:Xueshuantong injection+CT | 0 | No concerns | Low risk | No concerns | Major concerns | No concerns | Major concerns | Low |
| Shuxuetong injection+CT:Xueshuantong injection+CT | 0 | Some concerns | Low risk | No concerns | No concerns | No concerns | Major concerns | Very low |

7 Triglyceride

7.1 study limitations of the included studies


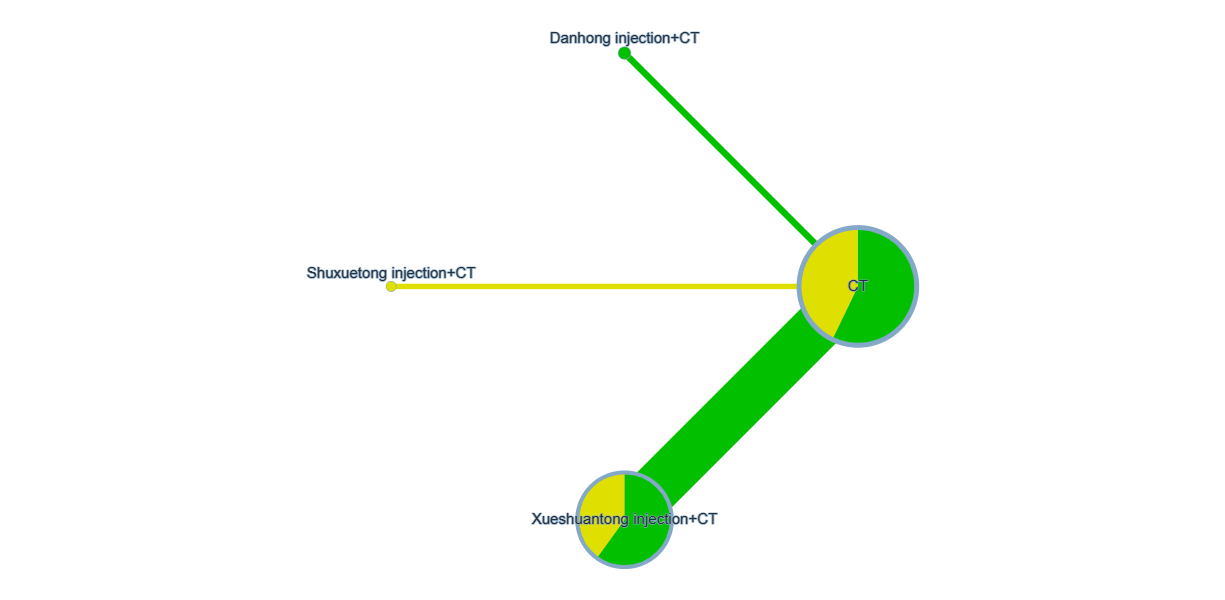


7.2 Contribution percentage of low, moderate, and high RoB comparisons to each network estimate

Low RoB is green, moderate RoB is yellow, high RoB is red.


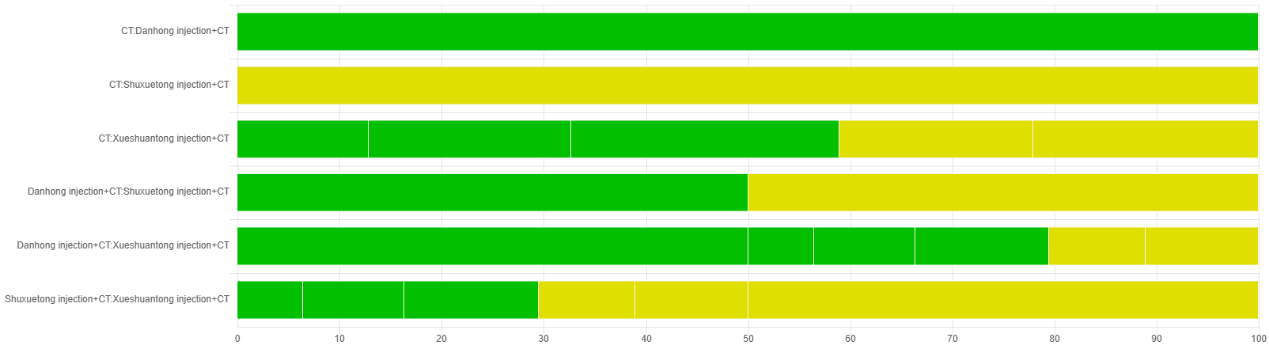


7.3 CINeMA for the primary outcome “triglyceride”

| Comparison | Number of studies | Within-study bias | Reporting bias | Indirectness | Imprecision | Heterogeneity | Incoherence | Confidence rating |
| --- | --- | --- | --- | --- | --- | --- | --- | --- |
| CT:Danhong injection+CT | 1 | No concerns | Low risk | No concerns | Major concerns | No concerns | Major concerns | Low |
| CT:Shuxuetong injection+CT | 1 | Some concerns | Low risk | No concerns | No concerns | No concerns | Major concerns | Very low |
| CT:Xueshuantong injection+CT | 5 | No concerns | Low risk | No concerns | No concerns | No concerns | Major concerns | Low |
| Danhong injection+CT:Shuxuetong injection+CT | 0 | Some concerns | Low risk | No concerns | No concerns | No concerns | Major concerns | Very low |
| Danhong injection+CT:Xueshuantong injection+CT | 0 | No concerns | Low risk | No concerns | No concerns | No concerns | Major concerns | Low |
| Shuxuetong injection+CT:Xueshuantong injection+CT | 0 | Some concerns | Low risk | No concerns | No concerns | No concerns | Major concerns | Very low |

8 Incidence of cerebral infarction

8.1 study limitations of the included studies


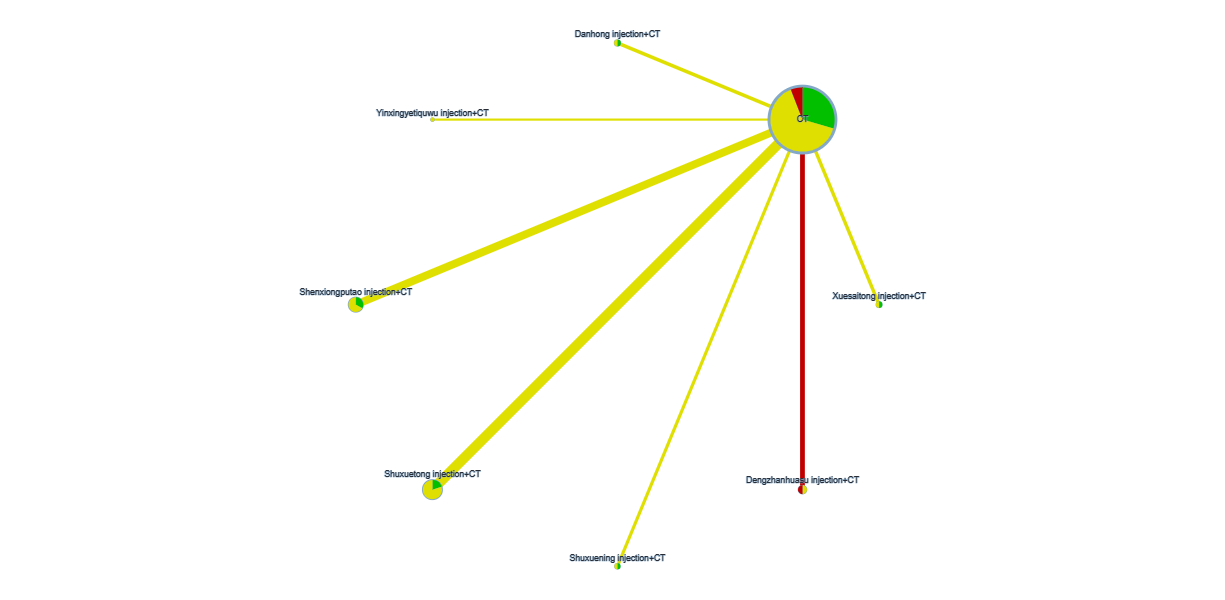


8.2 Contribution percentage of low, moderate, and high RoB comparisons to each network estimate

Low RoB is green, moderate RoB is yellow, high RoB is red.


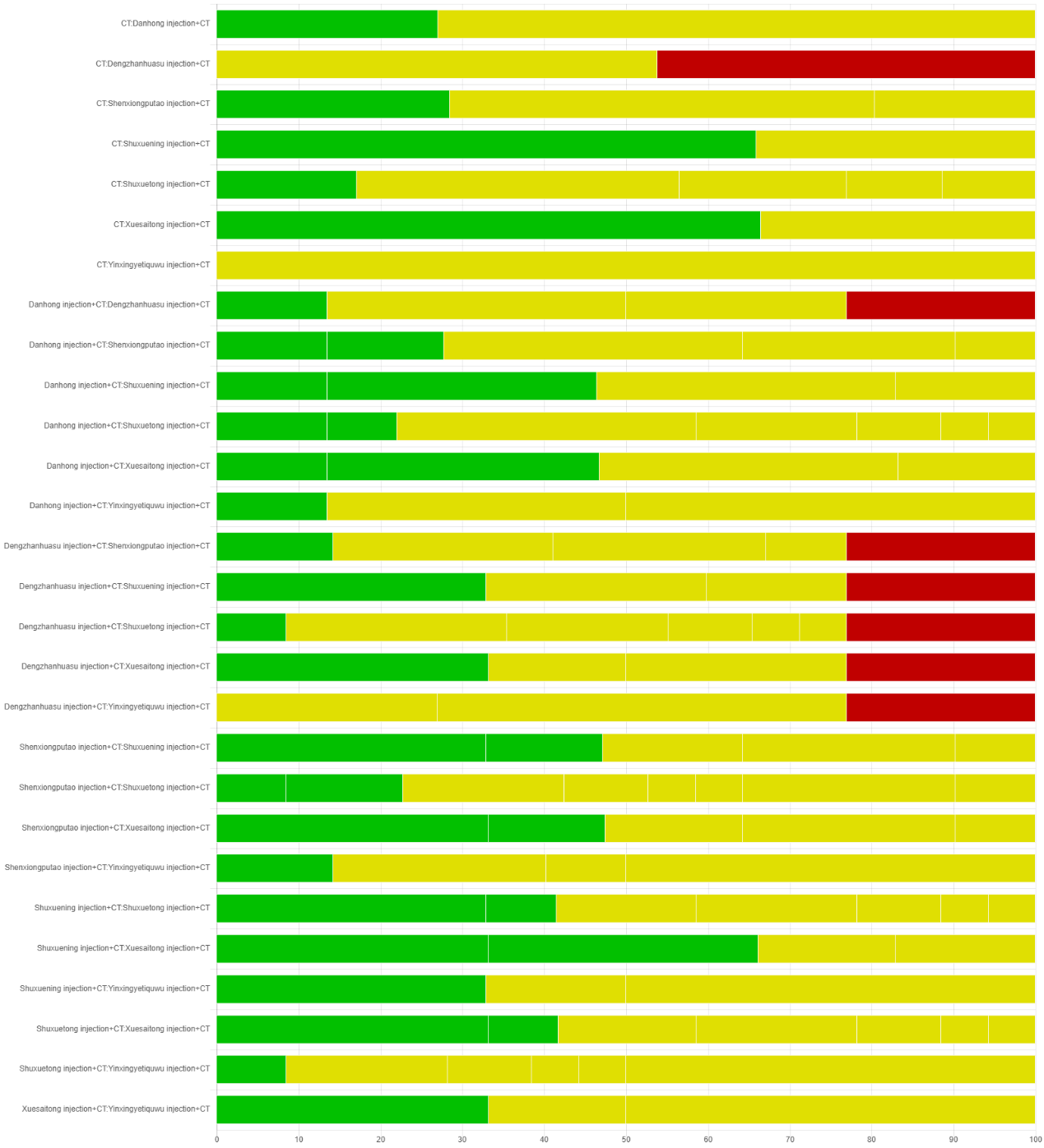


8.3 CINeMA for the primary outcome “Incidence of cerebral infarction”

| Comparison | Number of studies | Within-study bias | Reporting bias | Indirectness | Imprecision | Heterogeneity | Incoherence | Confidence rating |
| --- | --- | --- | --- | --- | --- | --- | --- | --- |
| CT:Danhong injection+CT | 2 | Some concerns | Low risk | No concerns | No concerns | No concerns | Major concerns | Very low |
| CT:Dengzhanhuasu injection+CT | 2 | Some concerns | Low risk | No concerns | Major concerns | No concerns | Major concerns | Very low |
| CT:Shenxiongputao injection+CT | 3 | Some concerns | Low risk | No concerns | Major concerns | No concerns | Major concerns | Very low |
| CT:Shuxuening injection+CT | 2 | No concerns | Low risk | No concerns | Major concerns | No concerns | Major concerns | Low |
| CT:Shuxuetong injection+CT | 5 | Some concerns | Low risk | No concerns | No concerns | No concerns | Major concerns | Very low |
| CT:Xuesaitong injection+CT | 2 | No concerns | Low risk | No concerns | Major concerns | No concerns | Major concerns | Low |
| CT:Yinxingyetiquwu injection+CT | 1 | Some concerns | Low risk | No concerns | Major concerns | No concerns | Major concerns | Very low |
| Danhong injection+CT:Dengzhanhuasu injection+CT | 0 | Some concerns | Low risk | No concerns | Major concerns | No concerns | Major concerns | Very low |
| Danhong injection+CT:Shenxiongputao injection+CT | 0 | Some concerns | Low risk | No concerns | Major concerns | No concerns | Major concerns | Very low |
| Danhong injection+CT:Shuxuening injection+CT | 0 | Some concerns | Low risk | No concerns | Major concerns | No concerns | Major concerns | Very low |
| Danhong injection+CT:Shuxuetong injection+CT | 0 | Some concerns | Low risk | No concerns | Major concerns | No concerns | Major concerns | Very low |
| Danhong injection+CT:Xuesaitong injection+CT | 0 | Some concerns | Low risk | No concerns | Major concerns | No concerns | Major concerns | Very low |
| Danhong injection+CT:Yinxingyetiquwu injection+CT | 0 | Some concerns | Low risk | No concerns | Major concerns | No concerns | Major concerns | Very low |
| Dengzhanhuasu injection+CT:Shenxiongputao injection+CT | 0 | Some concerns | Low risk | No concerns | Major concerns | No concerns | Major concerns | Very low |
| Dengzhanhuasu injection+CT:Shuxuening injection+CT | 0 | Some concerns | Low risk | No concerns | Major concerns | No concerns | Major concerns | Very low |
| Dengzhanhuasu injection+CT:Shuxuetong injection+CT | 0 | Some concerns | Low risk | No concerns | Major concerns | No concerns | Major concerns | Very low |
| Dengzhanhuasu injection+CT:Xuesaitong injection+CT | 0 | Some concerns | Low risk | No concerns | Major concerns | No concerns | Major concerns | Very low |
| Dengzhanhuasu injection+CT:Yinxingyetiquwu injection+CT | 0 | Some concerns | Low risk | No concerns | Major concerns | No concerns | Major concerns | Very low |
| Shenxiongputao injection+CT:Shuxuening injection+CT | 0 | Some concerns | Low risk | No concerns | Major concerns | No concerns | Major concerns | Very low |
| Shenxiongputao injection+CT:Shuxuetong injection+CT | 0 | Some concerns | Low risk | No concerns | Major concerns | No concerns | Major concerns | Very low |
| Shenxiongputao injection+CT:Xuesaitong injection+CT | 0 | Some concerns | Low risk | No concerns | Major concerns | No concerns | Major concerns | Very low |
| Shenxiongputao injection+CT:Yinxingyetiquwu injection+CT | 0 | Some concerns | Low risk | No concerns | Major concerns | No concerns | Major concerns | Very low |
| Shuxuening injection+CT:Shuxuetong injection+CT | 0 | Some concerns | Low risk | No concerns | Major concerns | No concerns | Major concerns | Very low |
| Shuxuening injection+CT:Xuesaitong injection+CT | 0 | No concerns | Low risk | No concerns | Major concerns | No concerns | Major concerns | Low |
| Shuxuening injection+CT:Yinxingyetiquwu injection+CT | 0 | Some concerns | Low risk | No concerns | Major concerns | No concerns | Major concerns | Very low |
| Shuxuetong injection+CT:Xuesaitong injection+CT | 0 | Some concerns | Low risk | No concerns | Major concerns | No concerns | Major concerns | Very low |
| Shuxuetong injection+CT:Yinxingyetiquwu injection+CT | 0 | Some concerns | Low risk | No concerns | Major concerns | No concerns | Major concerns | Very low |
| Xuesaitong injection+CT:Yinxingyetiquwu injection+CT | 0 | Some concerns | Low risk | No concerns | Major concerns | No concerns | Major concerns | Very low |
