# Supplementary material for: Comparative efficacy and safety of traditional Chinese medicine injections in patients with transient ischemic attack: A systematic review and network meta-analysis
Source: PLoS One. 2024 Jul 24;19(7):e0307663. doi: 10.1371/journal.pone.0307663 (PMC11268667; doi:10.1371/journal.pone.0307663)

**S8 File. Finding sources of heterogeneity using network meta-regression.**

**Table S8 Network meta-regression** **for highly heterogeneous outcomes**

| Outcomes | Shared beta (median and 95% CI) | | | | |
| --- | --- | --- | --- | --- | --- |
|  | year | sample | male | age | period |
| Plasma viscosity | -0.07 (-0.54; 0.64) | 0.18 (-1.31; 0.56) | 0.15 (-6.04; 0.63) | -0.23 (-0.69; 0.09) | -0.44 (-0.78; -0.09)* |
| Fibrinogen | -0.02 (-0.96; 0.80) | 0.02 (-0.63; 0.69) | 0.02 (-0.54; 0.58) | -0.52 (-1.14; 0.20) | -2.57 (-5.65; 0.86) |
| Whole blood reduced viscosity (high shear rate) | 0.25 (-0.47; 0.90) | 0.014 (-0.47; 0.52) | 0.002 (-0.50; 0.59) | 0.0007 (-0.47; 0.47) | -0.33 (-0.85; 0.14) |
| Whole blood reduced viscosity (low shear rate) | 0.31 (-2.68; 2.16) | 0.18 (-1.43; 2.10) | 0.07 (-1.22; 1.60) | 0.44 (-1.09; 1.89) | -0.98 (-2.27; 0.33) |

CI: Credible Interval; ⋇: Significant influence factors, 95% CI does not contain zero.

**Plasma viscosity**

**year**

When the model was adjusted for centering value, compared with the control group, the MD value did not change significantly, and the hierarchy from the unadjusted model retained.


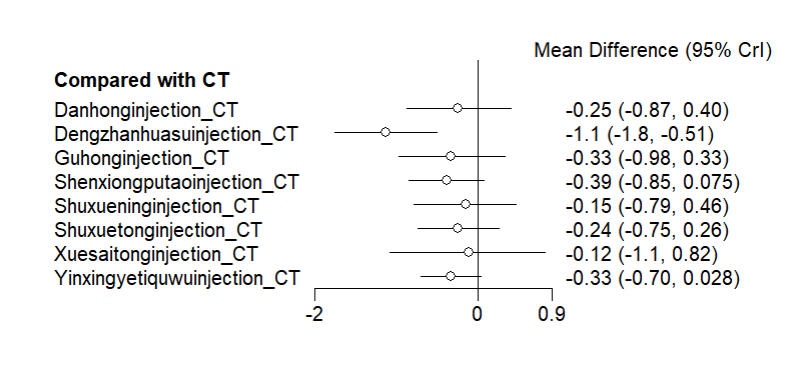


**sample**

When the model was adjusted for centering value, compared with the control group, the MD value did not change significantly, and the hierarchy from the unadjusted model retained.


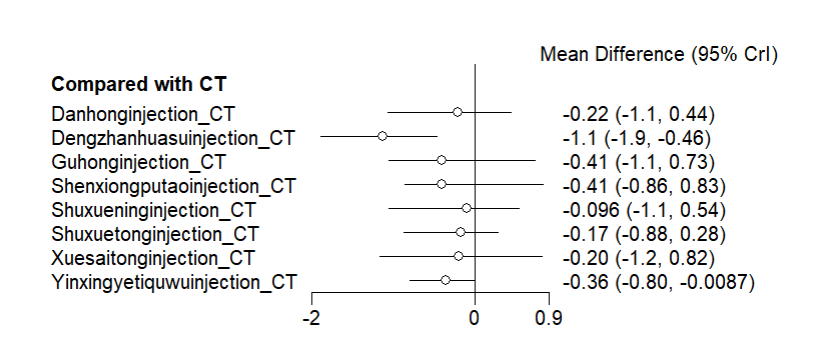


**male**

When the model was adjusted for centering value, compared with the control group, the MD value did not change significantly, and the hierarchy from the unadjusted model retained.


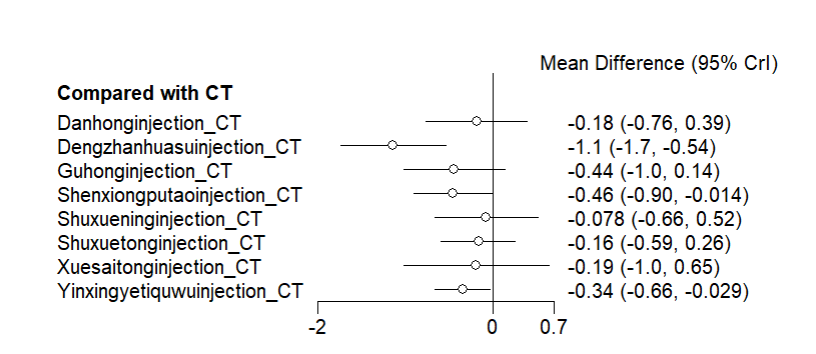


**age**

When the model was adjusted for centering value, compared with the control group, the MD value did not change significantly, and the hierarchy from the unadjusted model retained.


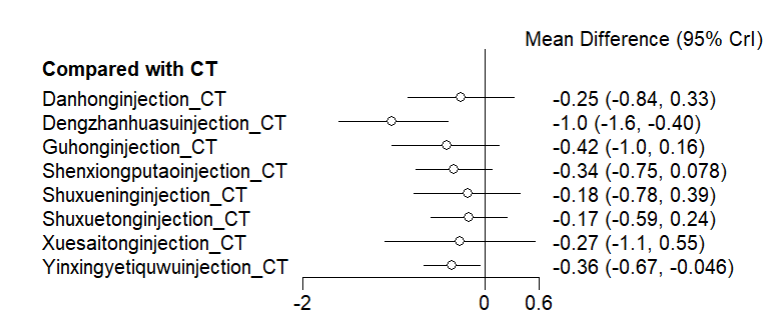


**Period**

When the model was adjusted for centering value, compared with the control group, the MD value did not change significantly, and the hierarchy from the unadjusted model retained.


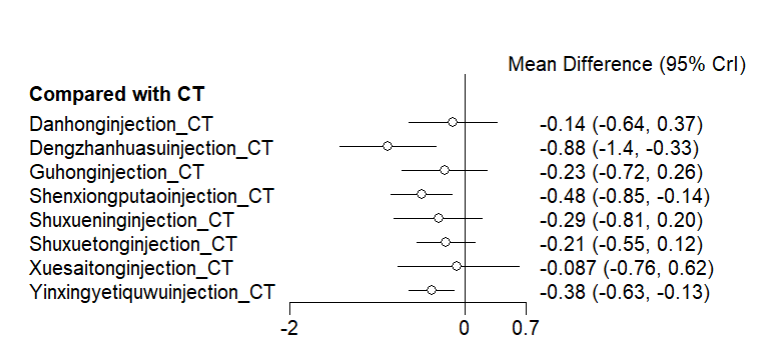


**Fibrinogen**

**year**

When the model was adjusted for centering value, compared with the control group, the MD value did not change significantly, and the hierarchy from the unadjusted model retained.


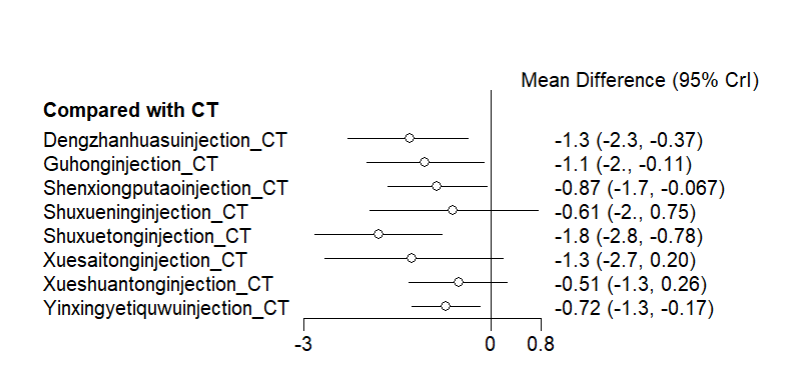


**Sample**

When the model was adjusted for centering value, compared with the control group, the MD value did not change significantly, and the hierarchy from the unadjusted model retained.


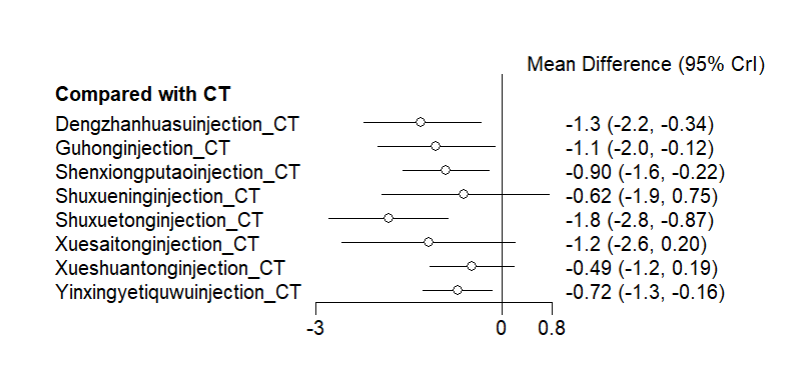


**male**

When the model was adjusted for centering value, compared with the control group, the MD value did not change significantly, and the hierarchy from the unadjusted model retained.


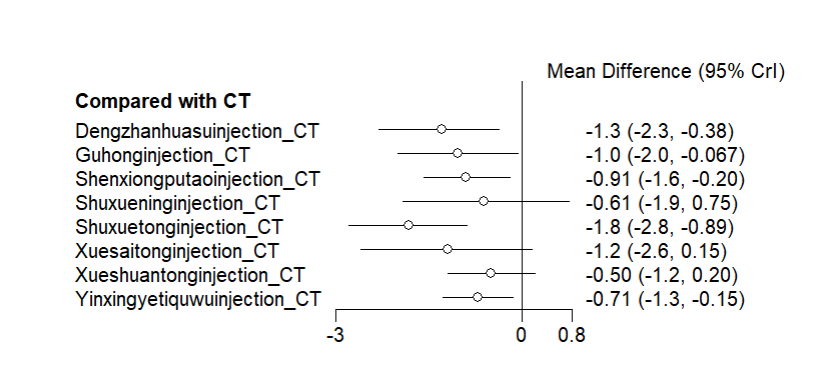
**age**

When the model was adjusted for centering value, compared with the control group, the MD value did not change significantly, and the hierarchy from the unadjusted model retained.


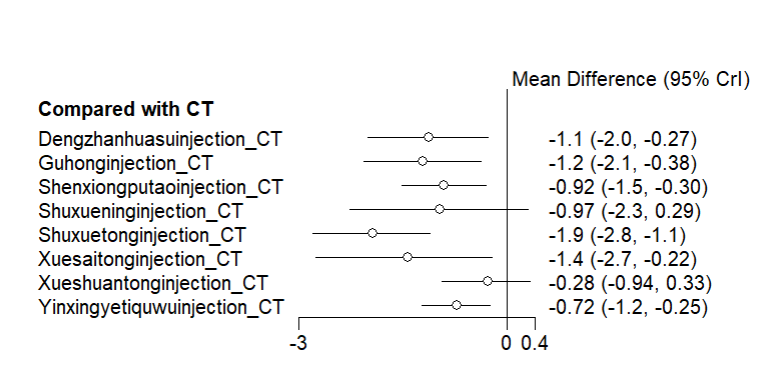


**Period**

When the model was adjusted for centering value , the MD value changed.


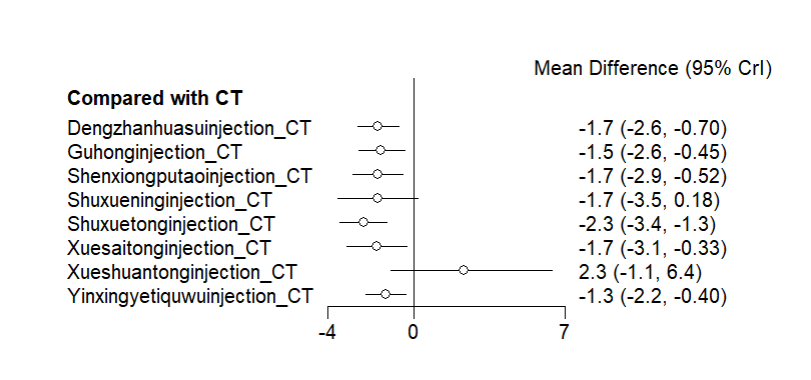


**Whole blood reduced viscosity (high shear rate)**

**year**

When the model was adjusted for centering value, compared with the control group, the MD value did not change significantly, and the hierarchy from the unadjusted model retained.


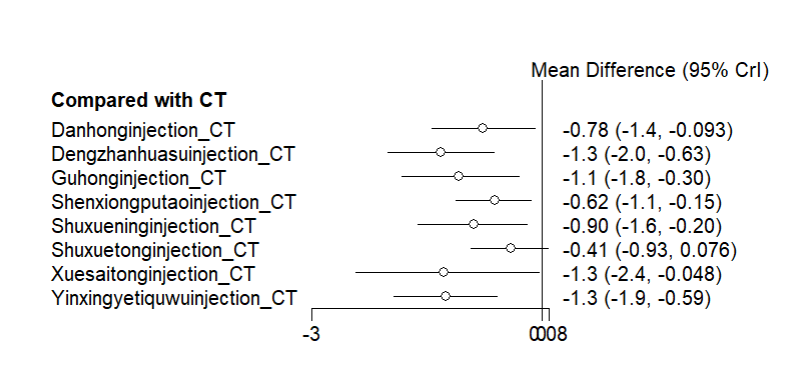


**sample**

When the model was adjusted for centering value, compared with the control group, the MD value did not change significantly, and the hierarchy from the unadjusted model retained.


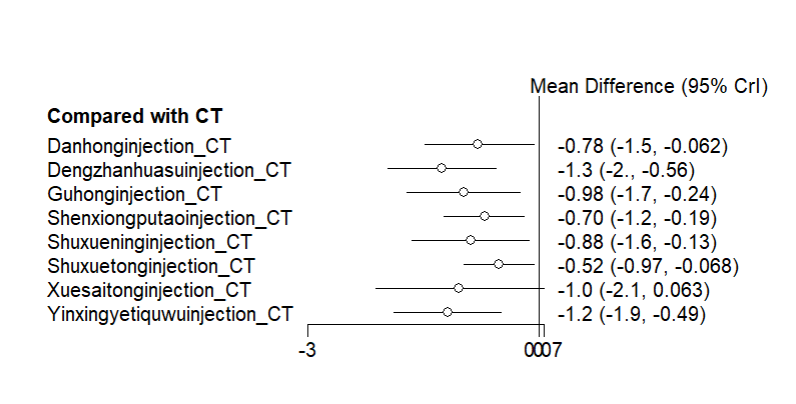


**male**

When the model was adjusted for centering value, compared with the control group, the MD value did not change significantly, and the hierarchy from the unadjusted model retained.


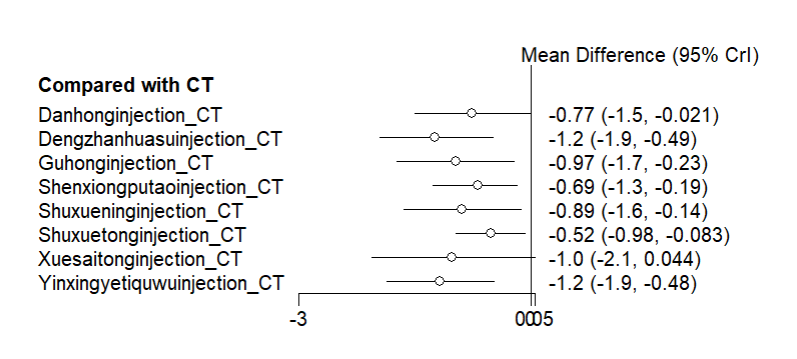


**age**

When the model was adjusted for centering value, compared with the control group, the MD value did not change significantly, and the hierarchy from the unadjusted model retained.


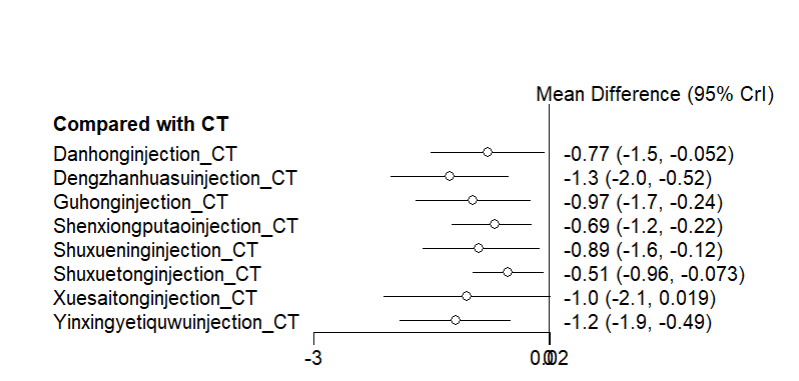


**Period**

When the model was adjusted for centering value, compared with the control group, the MD value did not change significantly, and the hierarchy from the unadjusted model retained.


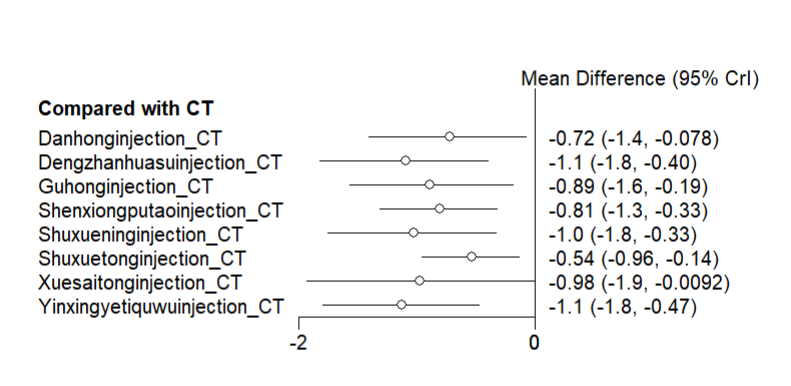


**Whole blood reduced viscosity (low shear rate)**

**Year**

When the model was adjusted for centering value, compared with the control group, the MD value did not change significantly, and the hierarchy from the unadjusted model retained.


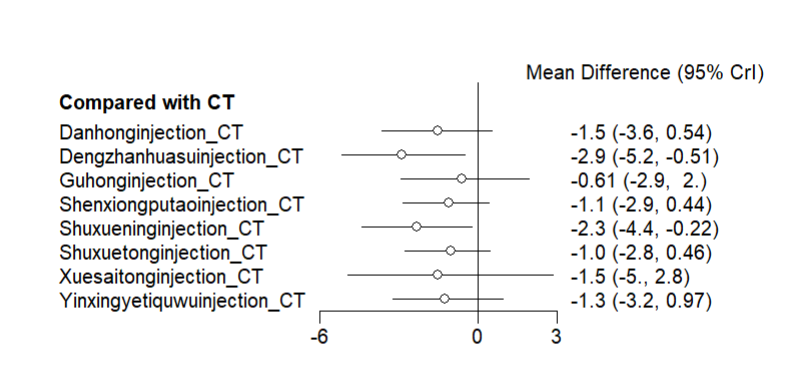


**sample**

When the model was adjusted for centering value, compared with the control group, the MD value did not change significantly, and the hierarchy from the unadjusted model retained.


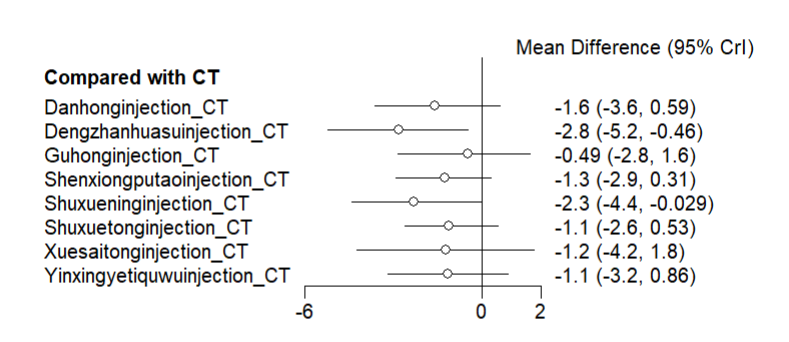


**male**

When the model was adjusted for centering value, compared with the control group, the MD value did not change significantly, and the hierarchy from the unadjusted model retained.


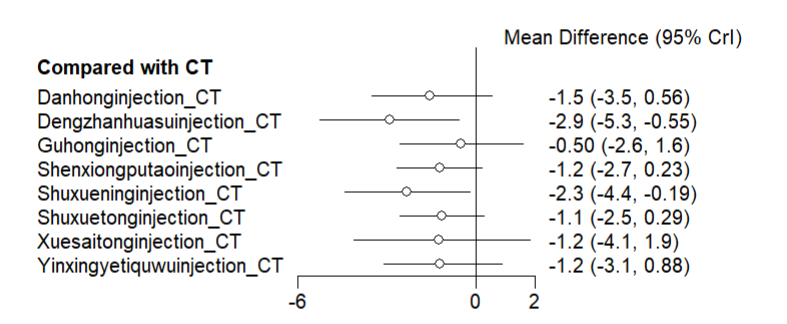


**age**

When the model was adjusted for centering value, compared with the control group, the MD value did not change significantly, and the hierarchy from the unadjusted model retained.


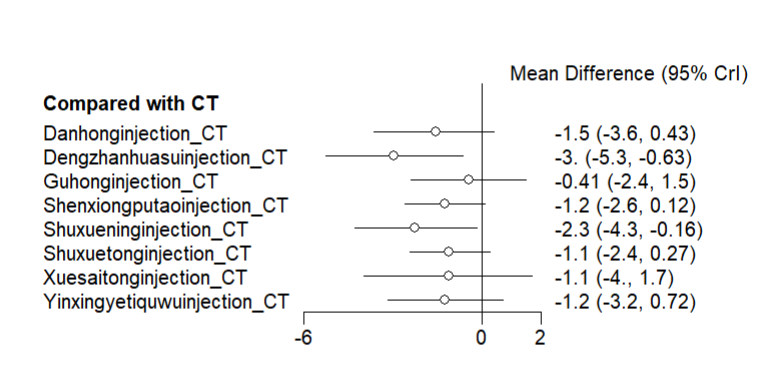


**Period**

When the model was adjusted for centering value, compared with the control group, the MD value did not change significantly, and the hierarchy from the unadjusted model retained.


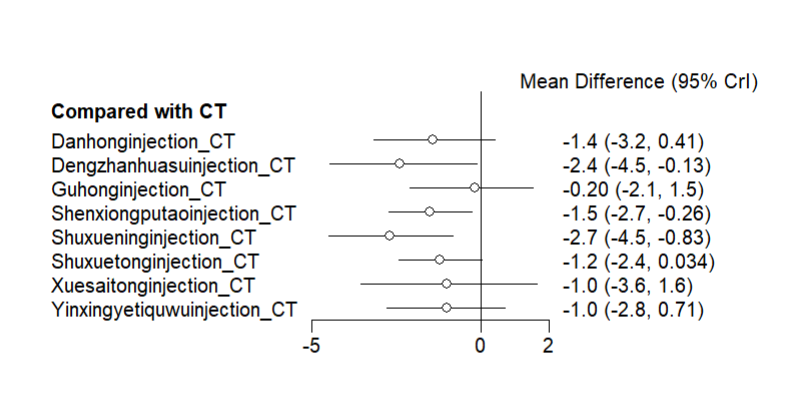

Supplement: S8 File — (DOCX) [file pone.0307663.s008.docx]
