# Supplementary material for: Comparative efficacy and safety of traditional Chinese medicine injections in patients with transient ischemic attack: A systematic review and network meta-analysis
Source: PLoS One. 2024 Jul 24;19(7):e0307663. doi: 10.1371/journal.pone.0307663 (PMC11268667; doi:10.1371/journal.pone.0307663)

**S9 File. Sensitivity analyses.**

**Table S9 Sensitivity analysis and heterogeneous changes.**

| **Including only studies with** | **Number of studies included** | **I^2^** | **Change** |
| --- | --- | --- | --- |
| **Total effectiveness rate** | | | |
| None | 48 | 0% | - |
| Sample size less than 70 | 39 | 0% | - |
| Less than 10 days and more than 20 days of treatment | 39 | 0% | - |
| **Plasma viscosity** | | | |
| None | 24 | 97.2% | - |
| Sample size less than 70 | 20 | 95.5% | -1.7% |
| Less than 10 days and more than 20 days of treatment | 19 | 97.2% | - |
| **Fibrinogen** | | | |
| None | 22 | 97.4% | - |
| Sample size less than 70 | 19 | 97.2% | -0.2% |
| Less than 10 days and more than 20 days of treatment | 15 | 98.2% | +0.8% |
| **Whole blood reduced viscosity (high shear rate)** | | | |
| None | 20 | 90.7% | - |
| Sample size less than 70 | 18 | 92.2% | +1.5% |
| Less than 10 days and more than 20 days of treatment | 16 | 93.2% | +2.5% |
| **Whole blood reduced viscosity (low shear rate)** | | | |
| None | 19 | 94.1% | - |
| Sample size less than 70 | 17 | 95.1% | +1.1% |
| Less than 10 days and more than 20 days of treatment | 15 | 94.6% | +0.5% |
| **Incidence of cerebral infarction** |  |  |  |
| None | 17 | 0% | - |
| Sample size less than 70 | 16 | 0% | - |
| Less than 10 days and more than 20 days of treatment | 13 | 0% | - |
| **Total cholesterol** |  |  |  |
| None | 8 | 0% | - |
| Sample size less than 70 | 5 | 0% | - |
| Less than 10 days and more than 20 days of treatment | 3 | NA | - |
| **Triglyceride** |  |  |  |
| None | 7 | 0.5% | - |
| Sample size less than 70 | 5 | 18% | +17.5% |
| Less than 10 days and more than 20 days of treatment | 2 | NA | - |

We excluded a small number of studies from sensitivity analyses using specific criteria. The mean sample size for this study was 95. we found that most studies had a sample size of 70 or more, so we excluded studies with a sample size of less than 70. And the percentage of studies with sample size less than 70 is relatively small among all studies. The duration of treatment in this study was mainly focused on the 2-week effect, so we excluded studies with very short and very long duration of treatment. Also, the percentage of excluded studies was relatively small.

1 Total effectiveness rate

1.1

There was no significant change in the grading from the original results.


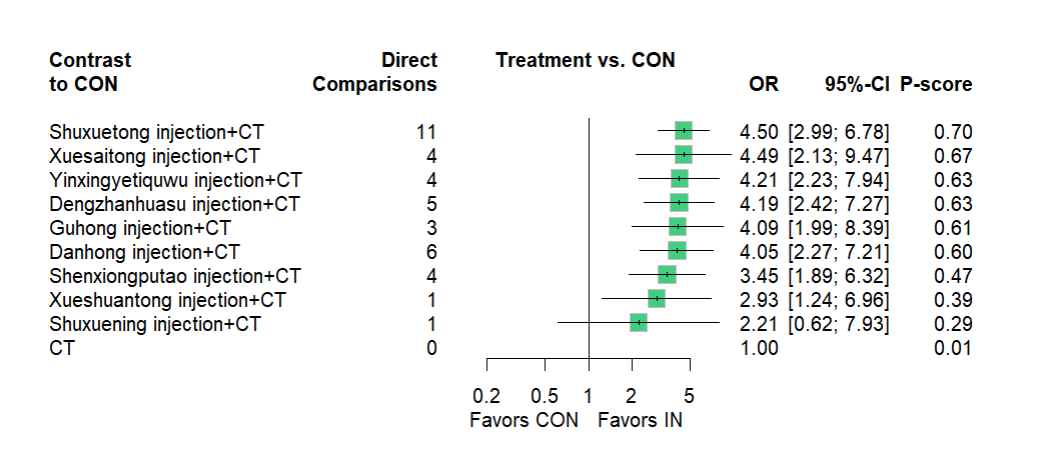


1.2

There was no significant change in the grading of most interventions compared to the original results.


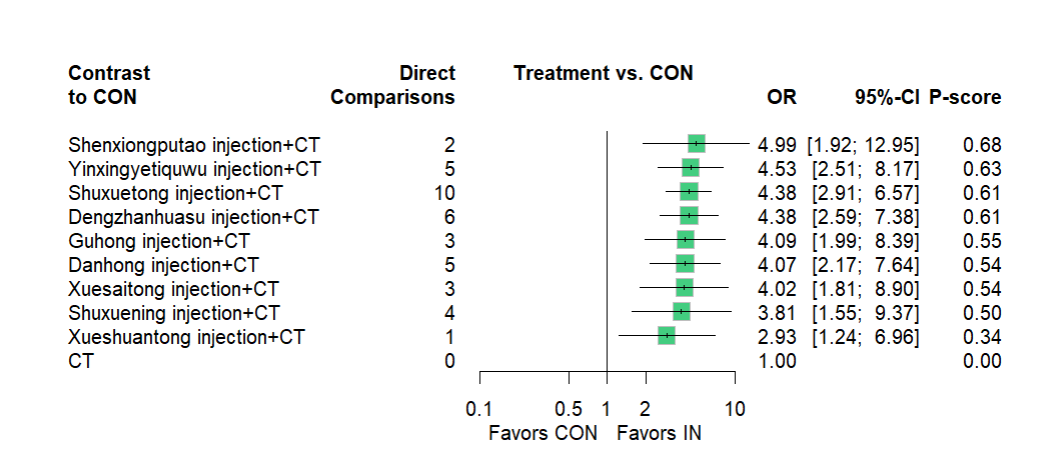


2 Plasma viscosity

2.1

P-score of Dengzhanhuasu injection + CT was significantly decreased and the rest of the results had no significant change in grade division compared to the original results.


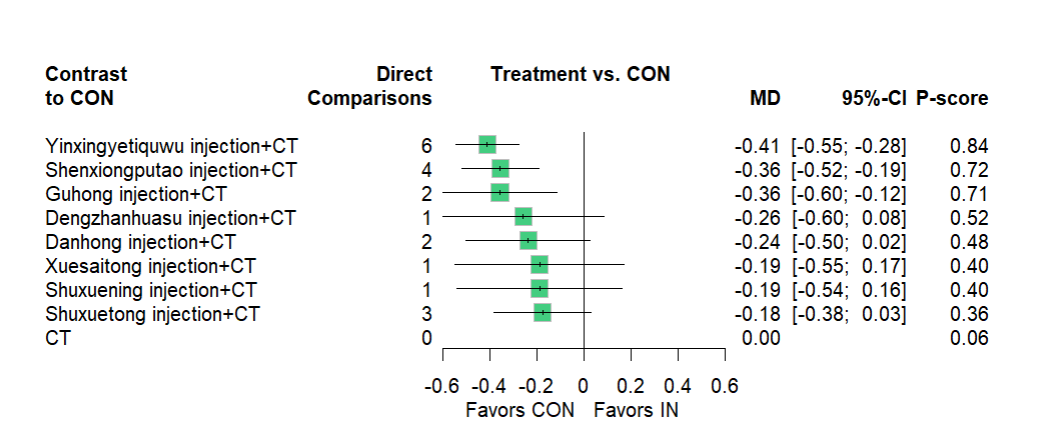


2.2

There was no significant change in the grading from the original results.


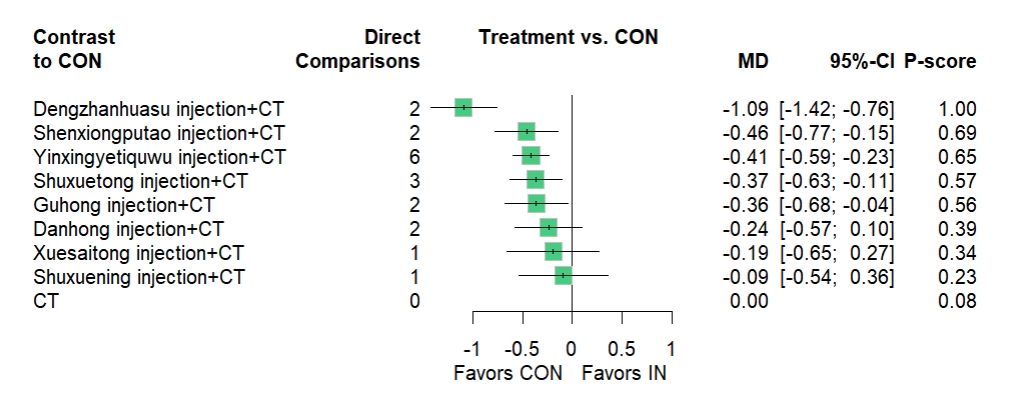


3 **Fibrinogen**

3.1

There was no significant change in the grading of most interventions compared to the original results.


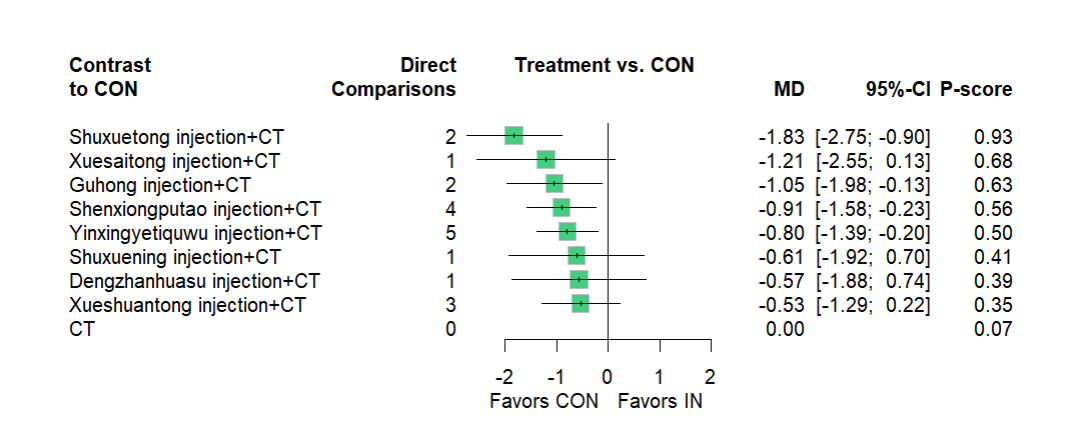


3.2 There was no significant change in the grading from the original results.


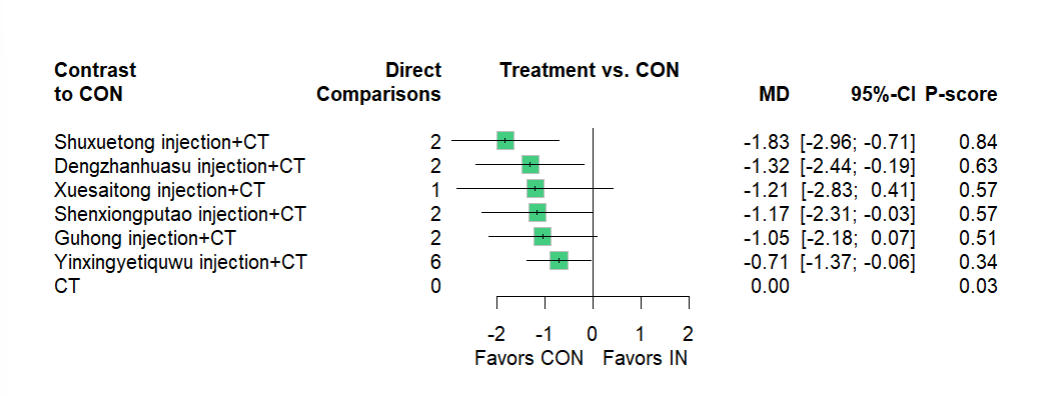


4 **Whole blood reduced viscosity (high shear rate)**

4.1

There was no significant change in the grading from the original results.


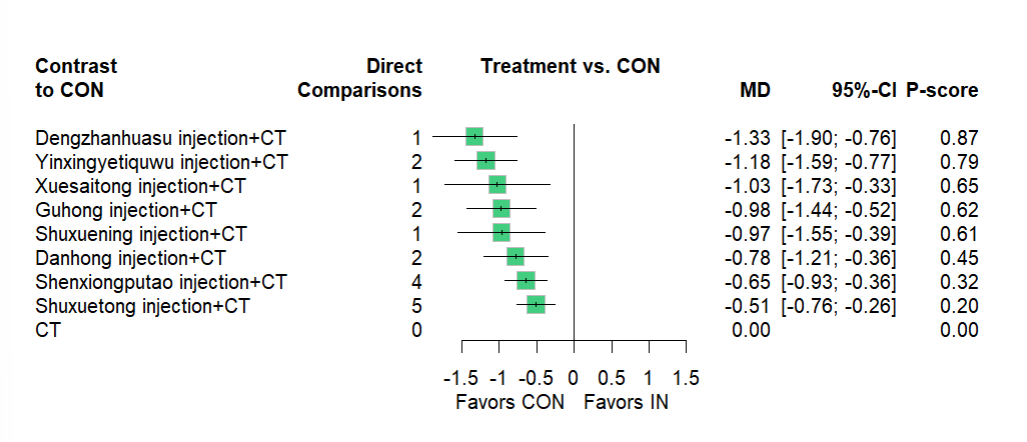


4.2

There was no significant change in the grading from the original results.


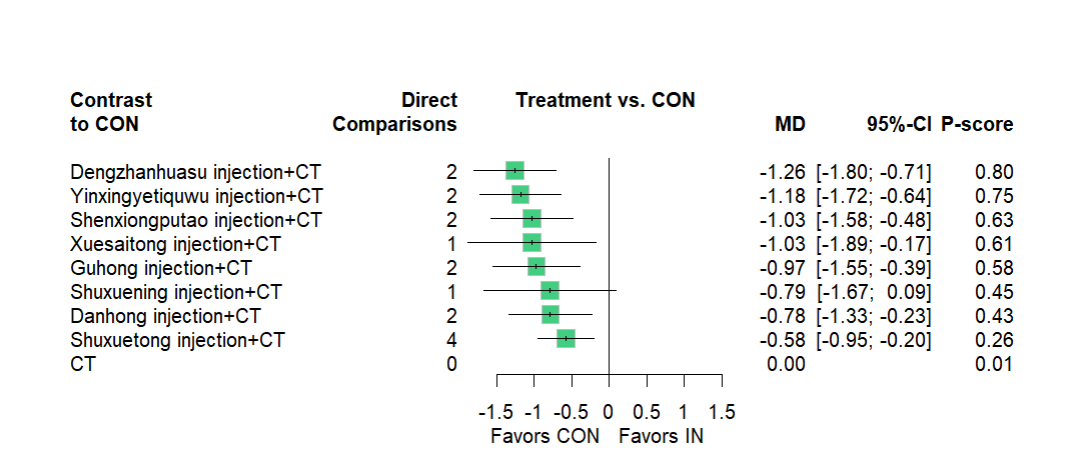


5 **Whole blood reduced viscosity (low shear rate)**

5.1

There was no significant change in the grading from the original results.


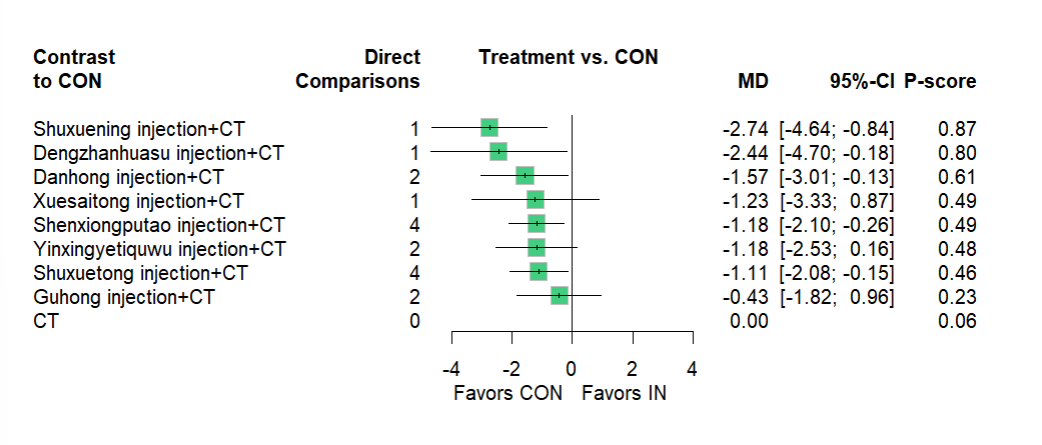


5.2

There was no significant change in the grading from the original results.


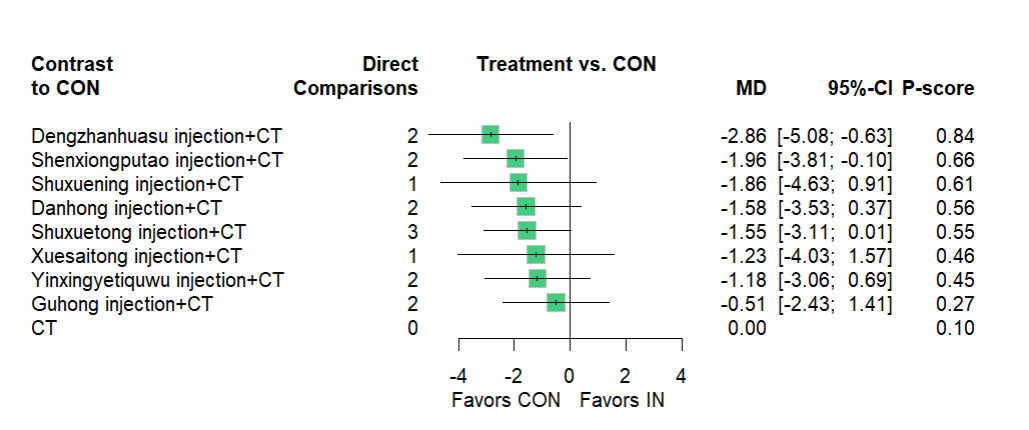


6 Incidence of cerebral infarction

6.1 There was no significant change in the grading from the original results.


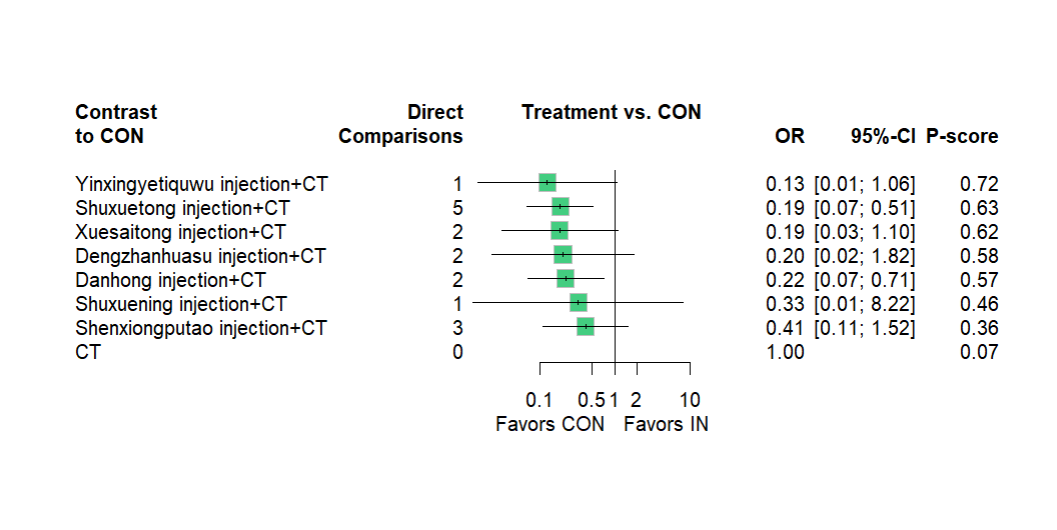


6.2

Only Shenxiongputao injection+CT was changed and ranked up.


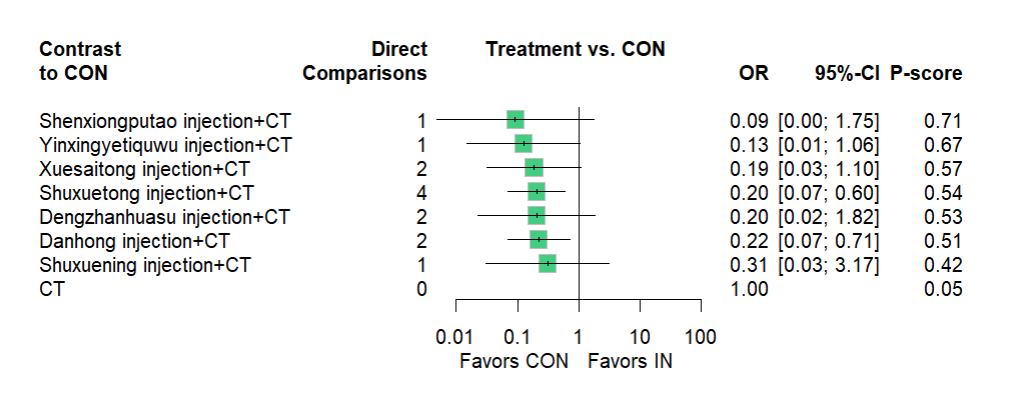


7 **T****otal cholesterol**

7.1

There was no significant change in the grading from the original results.


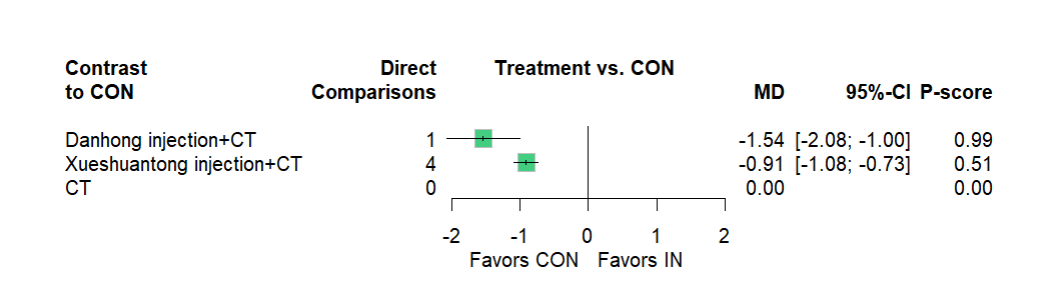


7.2

There was no significant change in the grading from the original results.


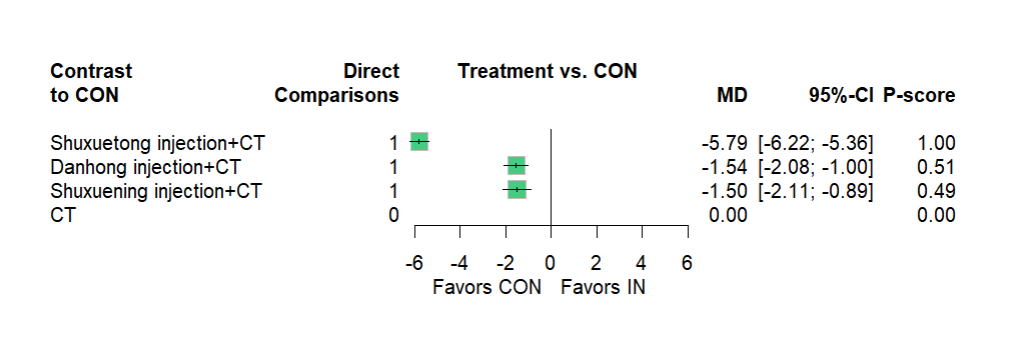


8 **Triglyceride**

8.1

There was no significant change in the grading from the original results.


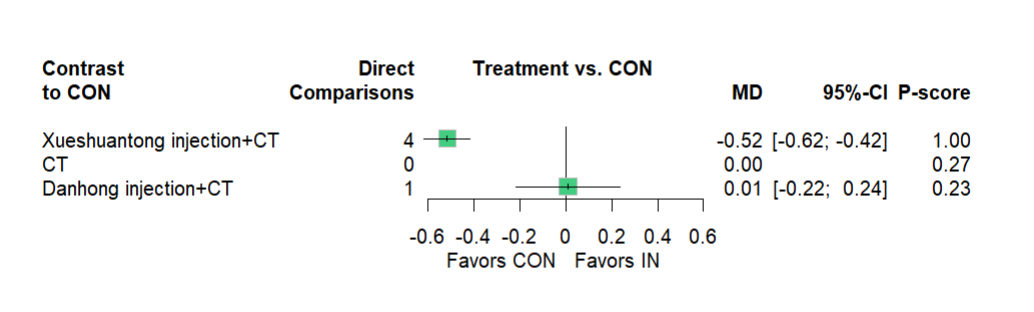


8.2

There was no significant change in the grading from the original results.


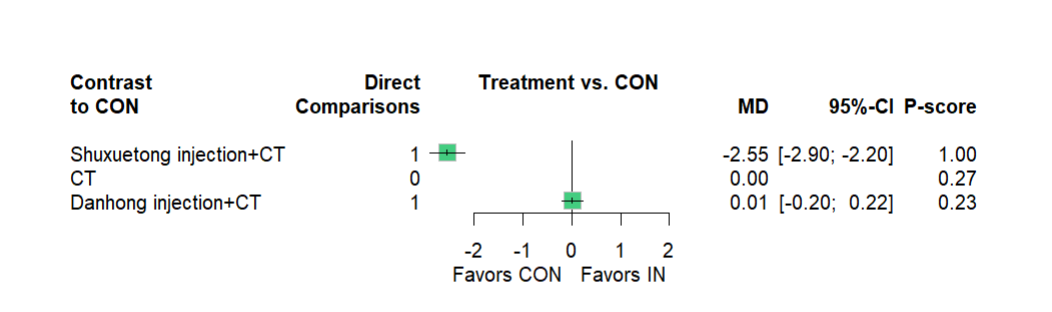

Supplement: S9 File — (DOCX) [file pone.0307663.s009.docx]
