# Supplementary material for: Comparative efficacy and safety of traditional Chinese medicine injections in patients with transient ischemic attack: A systematic review and network meta-analysis
Source: PLoS One. 2024 Jul 24;19(7):e0307663. doi: 10.1371/journal.pone.0307663 (PMC11268667; doi:10.1371/journal.pone.0307663)
Supplement: S10 File — (DOCX) [file pone.0307663.s010.docx]

**S10 File. Mechanism of action of traditional Chinese medicine injection.**

Table S10 Mechanism of action of Chinese medicine injection

| Injection | Ingredient | Machine | Efficacy | References |
| --- | --- | --- | --- | --- |
| Shuxuetong injection | leech | Reduced expression and activity of Fas, ACAT-2, HMGCR | Reduces synthesis and conversion of fatty acids and cholesterol | 83 |
|  |  |  | Antithrombotic, anticoagulant, anti-inflammatory | 92, 94 |
|  |  |  | Reduces TC and TG levels in patients with hyperlipidemia | 86 |
|  |  |  | Reduced IMT, plaque area and number | 90 |
|  | earthworm | Reduced serum and liver TC, TG and FFA levels | Reduces exogenous lipid absorption and endogenous lipid synthesis, regulates blood lipid level. | 85 |
|  |  |  | Antithrombotic, anticoagulant, anti-inflammatory | 91, 93 |
| Dengzhanhuasu injection | breviscapine | Activation of LKB1/AMPK pathway and inhibition of HMGCR levels | Improve the body's lipid metabolism level and reduce blood lipids | 96 |
|  |  |  | Inhibits platelet factor 3 (PF3) and coagulation factor V and significantly increases fibrinolytic activity | 98 |
|  |  | Reduced levels of IL-6, IL-1β, and TNF-α, oxidative stress indicator MDA | neuroprotection | 99 |
|  | Scutellarin | Regulation of Hippo-FOXO3A and PI3K/Akt signaling pathways | Inhibition of endothelial cell injury and apoptosis | 97 |
|  |  | Activated microglia | neuroprotection | 100 |
|  |  | Upregulation of eNOS expression and downregulation of VEGF, bFGF and iNOS expression | neuroprotection | 101 |
| Yinxingyetiquwu injection | ginkgo biloba extract | Antagonists of platelet-activating factorreceptors | Effectively inhibit platelet aggregation | 104 |
|  |  | Influence on cAMP, TxA(2) and Ca2+ metabolism in platelets | Inhibition of platelet activation | 105 |
|  |  | Decreases in MDA and ROS, decreases in TNF-α, IL-1α, 1L-6, and | Antioxidant and anti-inflammatory | 106 |
|  |  | Regulation TWEAK-Fn14 | Protecting the brain and heart from ischemia-reperfusion injury | 107 |
|  |  | Mediation of Akt/Nrf2 signaling pathway upregulates antioxidant protein levels of HO-1, Nqo1, SOD, p-Akt, p-Nrf2 and Nrf2 | Protects neurons from oxidative stress damage | 108 |
|  |  | Inhibits the production of pro-inflammatory cytokines IL-1β and TNF-α, but upregulates the production of anti-inflammatory cytokines IL-10 and IL-10R in the brain and reduces cholesterol aggregation in peripheral tissues. | Anti-inflammatory, protects vascular endothelial cells | 109, 110 |
|  |  |  | Reduces the size of infarcts caused by stroke, repairs neuronal damage, and reduces blood viscosity | 111, 112 |

A-cholesterol acyltransferase ACAT-2, Akt/nuclear factor-E2-related factor2 Akt/Nrf2, AMP-activated protein kinase AMPK, basic fibroblast growth factor bFGF, cyclic AMP cAMP, fatty acid synthase Fas, fibroblast growth factor-inducible 14 Fn14, Hippo-forkhead box O3A Hippo-FOXO3A, 3-hydroxy-3-methylglutaryl-coenzyme A reductase HMGCR, hemeoxygenase-1 HO-1, Liver kinase B1 LKB1, interleukin-1β IL-1β, intima-media thickness IMT, Malondialdehyde MDA, Nitric oxide synthase NOS, quinone oxidoreductase l Nqo1, nuclear factor-E2-related factor2 Nrf2, platelet factor 3 PF3, phosphorylated Akt p-Akt, phosphoinositide 3-kinase/Akt pathway PI3K/Akt, phosphorylated Nrf2 p-Nrf2, reactive oxygen species ROS, Superoxide Dismutase SOD, total cholesterol TC, triglyceride TG, tumor necrosis factor-α TNF-α, thromboxane A2 TxA2 , tumor necrosis factor-related weak inducer of apoptosis TWEAK, vascular endothelial growth factor VEGF.

10.1 Shuxuetong injection


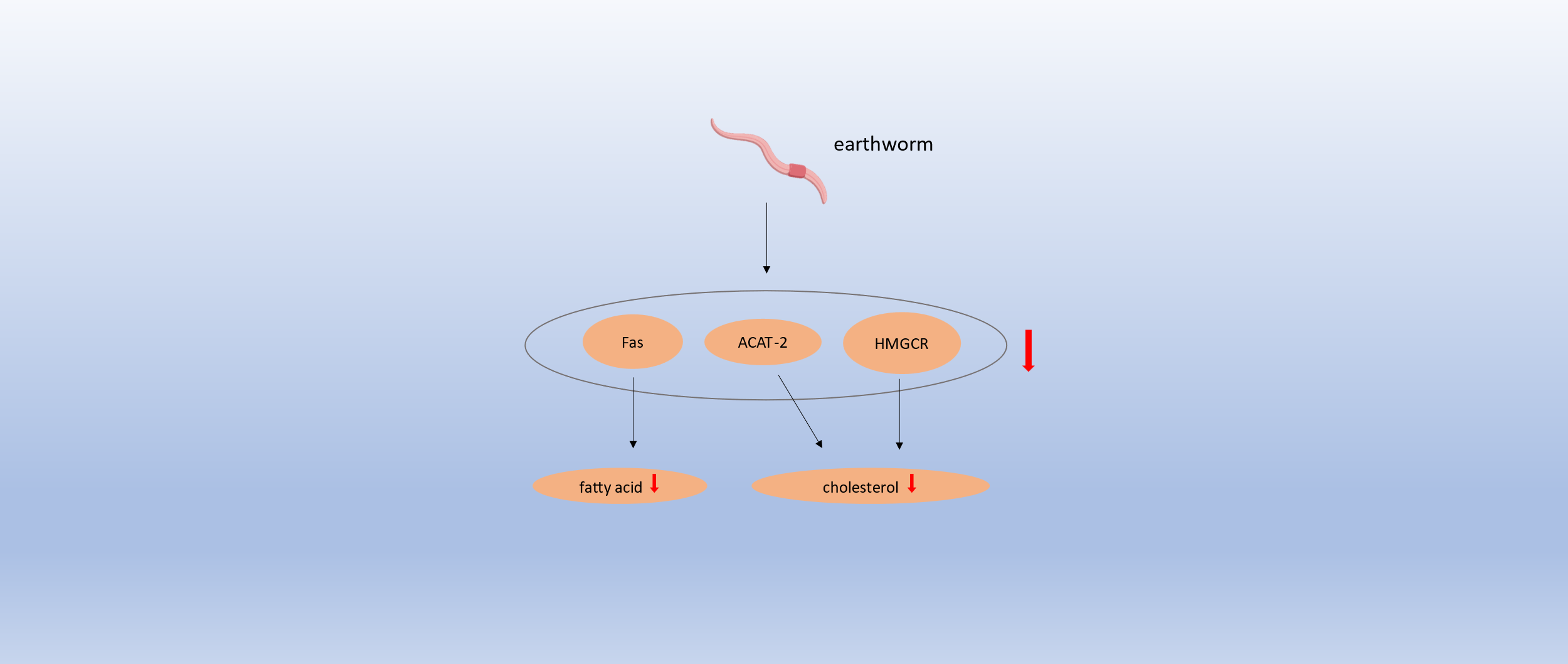


A-cholesterol acyltransferase ACAT-2, Fatty acid synthase Fas, 3-hydroxy-3-methylglutaryl-coenzyme A reductase HMGCR.

10.2 Dengzhanhuasu injection


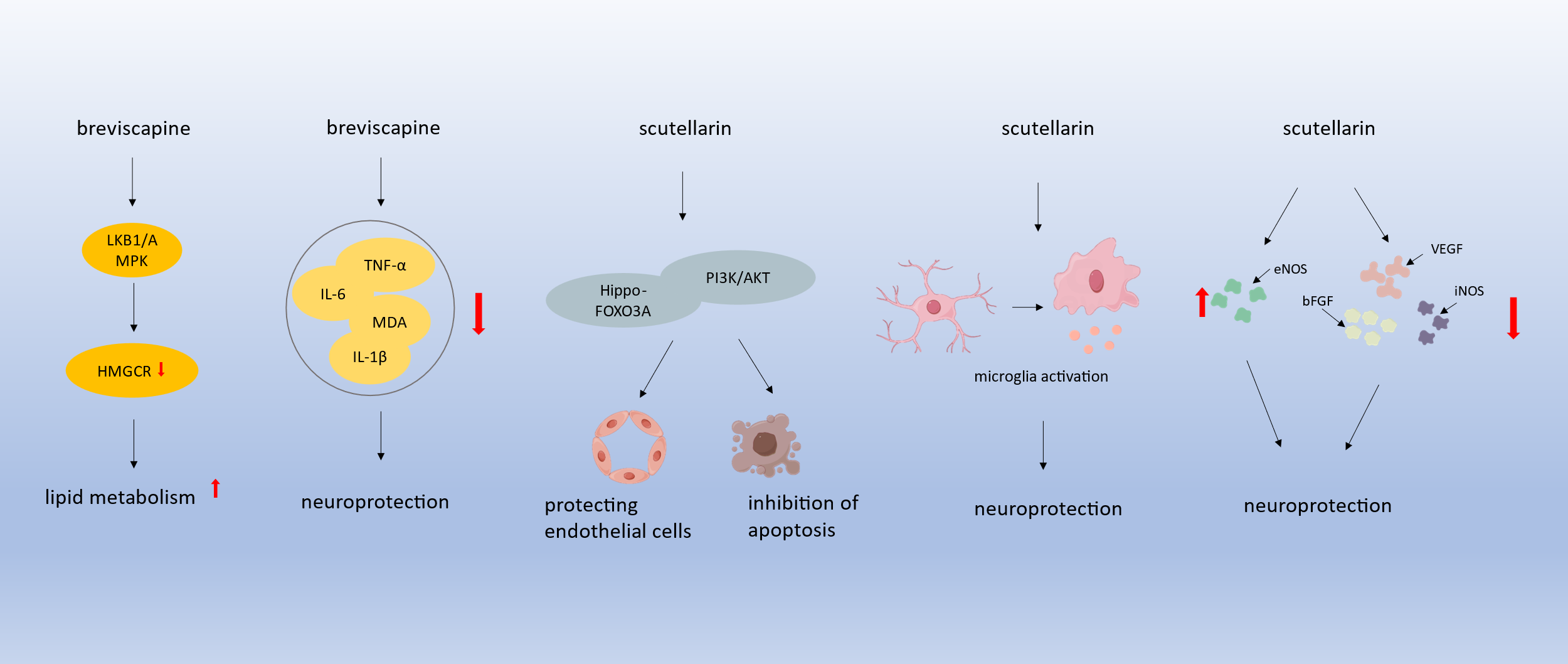


AMP-activated protein kinase AMPK, basic fibroblast growth factor bFGF, Hippo-forkhead box O3A Hippo-FOXO3A, 3-hydroxy-3-methylglutaryl-coenzyme A reductase HMGCR, Liver kinase B1 LKB1, interleukin-1β IL-1β, Malondialdehyde MDA, Nitric oxide synthase NOS, phosphoinositide 3-kinase/Akt pathway PI3K/Akt, tumor necrosis factor-α TNF-α, vascular endothelial growth factor VEGF.

10.3 Yinxingyetiquwu injection


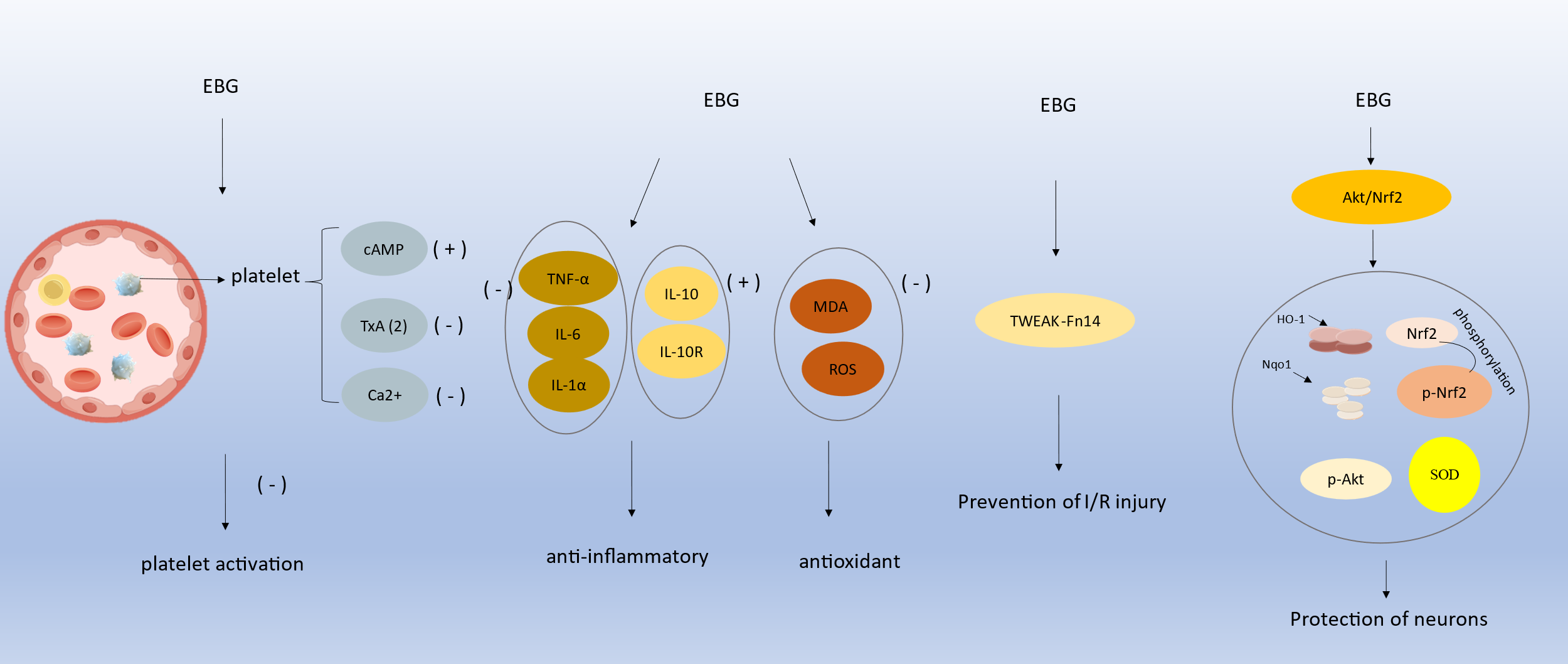


cyclic AMP cAMP, ginkgo biloba extract EBG, interleukin-1β IL-1β, ischemia/reperfusion I/R, Malondialdehyde MDA, nuclear factor-E2-related factor2 Nrf2, phosphorylated Akt p-Akt, Superoxide Dismutase SOD, tumor necrosis factor-α TNF-α, thromboxane A2 TxA2 , tumor necrosis factor-related weak inducer of apoptosis TWEAK.
